# Estrogen receptor alpha (ERα)–mediated coregulator binding and gene expression discriminates the toxic ERα agonist diethylstilbestrol (DES) from the endogenous ERα agonist 17β-estradiol (E2)

**DOI:** 10.1007/s10565-020-09516-6

**Published:** 2020-02-22

**Authors:** Aziza Hussein Bakheit Adam, Laura H. J. de Haan, Ignacio Miro Estruch, Guido J. E. J. Hooiveld, Jochem Louisse, Ivonne M. C. M. Rietjens

**Affiliations:** 1 Division of Toxicology, Wageningen University and Research, PO Box 8000, 6700 EA Wageningen, The Netherlands; 2 Division of Human Nutrition and Health, Wageningen University and Research, PO Box 17, 6700 AA Wageningen, The Netherlands

**Keywords:** Estrogen receptor alpha, Diethylstilbestrol, 17β-estradiol, Coregulator binding, Transcriptomics

## Abstract

Diethylstilbestrol (DES) is a synthetic estrogen and proven human teratogen and carcinogen reported to act via the estrogen receptor α (ERα). Since the endogenous ERα ligand 17β-estradiol (E2) does not show these adverse effects to a similar extent, we hypothesized that DES’ interaction with the ERα differs from that of E2. The current study aimed to investigate possible differences between DES and E2 using in vitro assays that detect ERα-mediated effects, including ERα-mediated reporter gene expression, ERα-mediated breast cancer cell (T47D) proliferation and ERα-coregulator interactions and gene expression in T47D cells. Results obtained indicate that DES and E2 activate ERα-mediated reporter gene transcription and T47D cell proliferation in a similar way. However, significant differences between DES- and E2-induced binding of the ERα to 15 coregulator motifs and in transcriptomic signatures obtained in the T47D cells were observed. It is concluded that differences observed in binding of the ERα with several co-repressor motifs, in downregulation of genes involved in histone deacetylation and DNA methylation and in upregulation of CYP26A1 and CYP26B1 contribute to the differential effects reported for DES and E2.

**Electronic supplementary material:**

The online version of this article (10.1007/s10565-020-09516-6) contains supplementary material, which is available to authorized users.

## Introduction

Diethylstilbestrol (DES) is a synthetic estrogen that has been used from the 1940s to the 1970s to prevent premature delivery and fetal death by stimulating the synthesis of estrogen and progesterone in the placenta (IARC 2012). In addition, DES was used in hormonal therapy applied for the treatment of prostate and breast cancer (Giusti et al. 1995; IARC 2012; Reed and Fenton 2013). From 1971 onwards, the use of DES was prohibited since it was shown to induce rare reproductive tract cancers in women exposed in utero, while no protective effect against miscarriage and premature delivery was actually observed (Titus-Ernstoff et al. 2001). Although DES has been discontinued since 1971, adverse health effects have later been discovered in women who had taken DES, as well as in their offspring including even subsequent generations. Adverse effects included breast cancer, clear cell adenocarcinoma of the vagina and cervix, abnormalities in the female genital tract and abnormalities of the male reproductive tract (Colton and Greenberg 1982; Palmer et al. 2006).

**Fig. 1:**
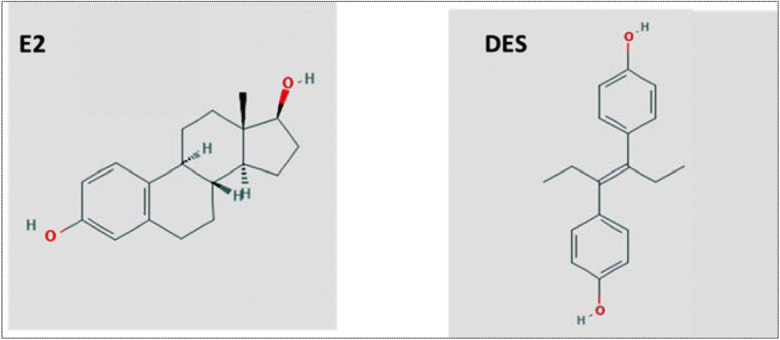
Chemical structures for E2 and DES

DES is an analogue of the endogenous female sex hormone 17β-estradiol (E2) and binds to both the estrogen receptor α (ERα) and estrogen receptor β (ERβ) (Bolger et al. 1998; Nikov et al. 2001). It has been reported that the molecular dimensions of DES are almost identical to those of E2, particularly with regard to the distance between the terminal hydroxyl groups (Gonzalez et al. 2019) (Fig. 1). Molecular docking of E2 and DES into the ligand binding domain of ERα from mouse and rat revealed similar binding orientations and confirmed a role for the hydroxyl moieties in this interaction (Gonzalez et al. 2019). The ERα agonist action has generally been associated with stimulation of cell proliferation, while ERβ activation has been linked with suppression of cell proliferation and stimulation of apoptosis (Sotoca et al. 2008; Thomas and Gustafsson 2011).

The mode of action by which DES causes its adverse effects has not been unraveled yet. It has been reported that the ERα is required in the mediation of the proliferative response to DES in uterus and prostate epithelial cells in vivo (Chen et al. 2012; Klotz et al. 2000). Several studies have indicated that a functional ERα is needed for DES-mediated adverse effects, including phenotypic changes in the reproductive tract and progressive proliferative lesions and abnormal epithelial cell differentiation in the prostate (Chen et al. 2012). This is apparent from studies in which these DES-induced adverse effects were observed in wild type mice, while the effects were absent in ERα knockout mice (Couse et al. 2001; Couse and Korach 2004; Prins et al. 2001). These studies suggest that DES elicits its adverse effects on the reproductive tract through an ERα-mediated mechanism. It is of interest to note that the endogenous ERα agonist E2 does not induce the adverse effects that have been reported for DES to a similar extent. This points at the possible existence of essential differences between ERα activation by DES on the one hand and E2 on the other hand. Such differences upon ERα binding may be due to possible differential recruitment of coregulators, including both coactivators that interact with receptors and enhance their activation, as well as co-repressors that interact with receptors and decrease their activation (Klinge 2000; McKenna et al. 1999).

So far, it has been reported that, in the presence of DES, the ERα interacts with coregulators NCOA1 (Nuclear receptor coactivator 1), NRIP1 (Nuclear receptor-interacting protein1) and PNRC2 (Proline-rich nuclear receptor coactivator 1), as indicated by binding to the coregulator motifs NCOA1_677_700, NRIP1_173_195 and PNRC2_118_139, respectively, using the MARCoNI (Microarray Assay for Real-time Coregulator-Nuclear receptor Interaction) technology (Wang et al. 2013). However, no extensive comparison has been carried out between the ERα-coregulator interactions in the presence of DES compared with E2. This raises the question to what extent DES-mediated coregulator recruitment to the ERα might be different from that of E2 and whether that could play a role in the differential biological effects of these two ERα agonists. The present study investigates the DES- and E2-dependent modulation of the interaction of ERα with coregulators using the MARCoNI technology and peptide microarrays containing 154 unique nuclear receptor coregulator motifs of 64 different coregulators. To provide further information on the possible differences between DES- and E2-induced ERα-mediated effects, the present study also assesses the relative potency of the two compounds as ERα agonists in a human osteosarcoma U2OS ERα reporter gene assay and in a proliferation assay of human ERα positive T47D breast cancer cells and quantifies DES- and E2-induced modulation of gene expression in T47D cells using next generation sequencing (RNA-seq) and transcriptome analysis.

## Materials and methods

### Cell lines and culture conditions

The U2OS (human osteosarcoma) cell line, stably expressing ERα in addition to a 3× estrogen-responsive element and TATA box binding protein combined with a luciferase gene (3x ERE-TATA-luciferase gene) was obtained from Biodetection Systems (BDS) (Amsterdam, The Netherlands). U2OS-ERα cells were grown in DMEM:F12, a 1:1 mixture of Dulbecco’s modified Eagle medium (DMEM) and Ham’s nutrient mixture F12 (Gibco, Bleiswijk, The Netherlands) supplemented with 10% fetal bovine serum (FBS, Sigma-Aldrich, St. Louis, Missouri, United States), 0.5% non-essential amino acids (NEAA) (Gibco, Bleiswijk, The Netherlands), 200 μ/ml geneticin G418 (Gibco, Bleiswijk, The Netherlands) and 50 μg/ml hygromycin (PAA Laboratories GmbH, Pasching, Austria). T47D cells, obtained from the American Type Culture Collection (Manasssaa, VA, USA), were grown in 1:1 DMEM:F12/Glutamax culture medium supplemented with 10% FCS. All cells were incubated at 37 °C and 5% CO_2_ in a humidified atmosphere.

The T47D cell line is a generally applied model for studying ERα-mediated effects especially because the cells retain several key characteristics specific to the mammary epithelium (Holliday and Speirs 2011). Given that the adverse effects of DES are mediated through the ERα (Couse et al. 2001; Couse and Korach 2004; Prins et al. 2001), the T47D model was considered suitable to study potential differences in ERα-mediated responses toward DES and E2.

The human breast cancer cell line MCF-7 (provided by the American Type Culture Collection (Manasssaa, VA, USA) was cultured in Dulbecco’s Modified Eagle Medium DMEM/F12 (Gibco, Bleiswijk, The Netherlands) supplemented with 10% (v/v) fetal bovine serum (PAA, Pasching, Austria), kept in a humidified atmosphere at 37 °C and 5% CO_2_ and subcultured when they reached 60–80% confluence.

### Reporter gene assay

U2OS-ERα cells were seeded in 96-well white plates (PerkinElmer, Groningen, The Netherlands) at a density of 10^5^ cells/ml in phenol red free medium (DMEM/F12) supplemented with DCC-FCS (dextran-coated charcoal-treated fetal calf serum obtained from Gibco (Bleiswijk, The Netherlands) adding 100 μl/well and the cells were incubated at 37 °C and 5% CO_2_ in a humidified atmosphere. Twenty-four hours after seeding, medium was changed to phenol red free medium. Forty-eight hours after seeding, cells were exposed to the test compounds in triplicate, in phenol red free medium (DMEM/F12) supplemented with DCC-FCS. Exposure medium was prepared to reach the final concentration range of 0.1–100 pM for both DES (Sigma-Aldrich, Zwijndrecht, The Netherlands) and E2 (Sigma-Aldrich) using 200-time concentrated stock solution in DMSO (Acros, Geel, Belgium) diluted in the culture medium. The maximum concentration of DMSO in exposure medium was 0.5%. After removing the medium from the wells, 100 μL of exposure medium containing the test compound were added to the wells and the cells were incubated for another 24 h at 37 °C and 5% CO_2_ in a humidified atmosphere. After 24 h of exposure, cells were washed with 0.5× PBS and lysed with 30 μl of hypotonic low-salt buffer containing 10 mM Tris, 2 mM dithiothreitol (DTT, Sigma-Aldrich) and 2 mM 1,2-diaminocyclohexane tetraacetic acid monohydrate (CDTA, Sigma-Aldrich) pH 7.8. Plates were kept on ice for at least 30 min and subsequently stored at − 80 °C until analysis. One hour before measurement, plates were thawed on a plate shaker until they reached room temperature. Luciferase activity was determined using a luminometer (GloMax, Promega Corporation, USA). Data from the U2OS-ERα reporter gene assay were expressed in relative luminescence units (RLU), corrected for the corresponding background signal measured before luciferin induction. Effects obtained in the studies were expressed as a percentage of the maximum response obtained for E2 set at 100%.

### Cell proliferation

T47D cells were seeded in 96-well plates (Corning, NY, USA) at a cell density of 5 × 10^3^ cells/well in phenol red free medium (DMEM/F12) supplemented with DCC-FCS and incubated at 37 °C and 5% CO_2_ in a humidified atmosphere. Cells were allowed to attach and 24 h later exposed to the test compounds (1–10,000 pM for both E2 and DES, final solvent control 0.5% DMSO). After exposure for 72 h, 20 μl BrdU labelling solution (containing 5-bromo-2′-deoxyuridine in PBS) diluted (10% *v*/v) in exposure medium were added to the cells during the last 4 h of exposure. Next, BrdU incorporation was measured by fixation-denaturation of the cells followed by incubation with BrdU detection antibodies and the corresponding substrate according to the manufacturer’s guidelines (Roche, Manheim, Germany). Subsequently, colorimetric measurements were carried out at a wavelength of 370 nm with a Microplate Reader SpectraMax M2 (Molecular Devices, Sunnyvale CA, USA). Effects obtained were expressed as percentage of the maximum response obtained for E2 set at 100%.

### Coregulator binding assay

Ligand-modulated interaction of coregulators with ERα was assessed using a PamChip peptide microarray with 154 coregulator motifs of 66 different coregulators (PamGene International BV, Den Bosch, The Netherlands). Briefly, all incubations were performed on a PamStation (PamGene) at 20 °C using two cycles per minute, as described by Wang et al. (2013). Polyhistidine (His) tagged ERα ligand binding domain (amino acids 302–552, partly purified from *Escherichia coli* (*E. coli*) (Qiagen, Germantown, MD, USA, final concentration 10 nM) and His antibody penta-His Alexa Fluor 488 conjugate (Qiagen, Germantown, MD, USA, final concentration 25 nM) were diluted in time-resolved fluorescence resonance energy transfer (TR-FRET) reaction buffer containing 20 mM Tris–HCl pH 7.5 (Tris: Sigma-Aldrich) (HCl: Merck, Darmstadt, Germany), 500 mM NaCl (Merck), 0.2% bovine serum albumin (BSA, Merck) and 0.05% Tween 20 (Bio-Rad, Veenendaal, The Netherlands). All mixtures were kept on ice until transferred to the PamChip microarrays. The test compounds were pre-dissolved in 50-time concentrated stock solutions in DMSO. The final concentrations of the test compounds ranged between 10^−12^ and 10^−5^ M, and the final DMSO concentration was 2%. A reaction mixture with 2% DMSO served as negative control. Each array was blocked for 20 cycles using 25 μl of blocking buffer (Tris-buffered saline) (TBS) (Bio-Rad) supplemented with 0.01% Tween 20 (Bio-Rad) and 1% BSA. Later, the blocking buffer was removed by aspiration, and the reaction mixture containing the test compound at the required concentration was added to the PamChip microarray in a final volume of 25 μl. This reaction-ligand mixture was incubated (pumped up and down the porous microarray membrane containing the 154 different coregulator motifs) for 80 cycles. Subsequently, unbound receptor was removed by washing the arrays with 25 μl TBS, and, finally, a tiff image of each array was acquired by the charge-coupled device (CCD) camera of the PamStation. Image analysis was performed using BioNavigator software (Version 62, PamGene International BV). Per array, the fluorescent signal of each spot, representative of ER binding to that particular coregulator motif, was quantified. For each spot, the binding signal as median fluorescence signal minus background for each peptide was calculated. The modulation index (MI) for a saturating concentration was subsequently determined by calculating the compound-induced log10-fold change of fluorescence in the presence of ligand over that in the presence of solvent only. As each array contains 154 unique coregulator motifs, each compound was characterized by a 154-point MI profile.

### Next generation sequencing (RNA-seq) and transcriptome analysis

T47D cells were seeded in 25-cm^2^ flasks (Corning, NY, USA) at a density of 10^5^ cells/ml. Twenty-four hours after seeding, medium was changed for phenol red free medium supplemented with DCC-FCS. Forty-eight hours after seeding, cells were treated with 10 nM E2, 10 nM DES, or control (0.5% DMSO) in duplicate, in phenol red free medium supplemented with DCC-FCS for 6 h. Next, cells were lysed, and total RNA was extracted and purified with the Quick RNA Miniprep kit (Zymo Research, Irvine, CA, USA) according to manufacturer’s guidelines. For quality control, spectrophotometric analysis using a Nanodrop (ND-1000 Thermoscientific Wilmington, Delaware, USA) and RIN analysis 2100 Bioanalizer (Agilent Technologies California, EE. UU) were utilized. Only samples with RNA integrity number (RIN) values higher than 8 were accepted for analysis. RNA-seq library preparation and sequencing was commissioned to BaseClear BV (Leiden, The Netherlands). Briefly, strand-specific messenger RNA sequencing libraries for the Illumina (San Diego, CA, USA) platform were generated, multiplexed, clustered, and sequenced on an Illumina HiSeq 2500 with a single-read 50-cycle sequencing protocol (15 million reads per sample).

Colorimetric sequencing signals were translated into base calls using internal Illumina software (CASAVA). Subsequently, using the tool bcl2fastq2 (version 2.18), the per-cycle basecall (BCL) files were demultiplexed and converted into per-read FASTQ sequence files for downstream analysis. Next, reads containing PhiX control signal were removed by BaseClear BV using an in-house filtering protocol. In addition, reads containing (partial) adapters were clipped (up to minimum read length of 50 bp). Finally, the quality of the FASTQ sequences was assessed by the tool FastQC (Andrews 2018) (version 0.11.5) and enhanced by trimming off low-quality bases by setting the cut-off of the error probability limit of the modified-Mott algorithm (Ewing and Green 1998) to 0.02.

The RNA-seq reads were then used to quantify transcript abundances. To this end, the tool Salmon (Patro et al. 2017) (version 0.8.2) was used to map the reads to the GRCh38.p10 genome assembly-based transcriptome sequences as annotated by the Ensembl genome database project (Zerbino et al. 2018) (Ensembl release v90). The obtained transcript abundance estimates and lengths were then imported in R using the package tximport (Soneson et al. 2015) (version 1.6.0) and summarized on the gene-level. Differential gene expression was determined using the package edgeR (Robinson et al. 2010) (version 3.20.5) utilizing the obtained estimated gene-level counts and offsets based on the transcript-level abundance estimates. The latter corrects for changes to the average transcript length across samples, and incorporation of such offsets has been reported to improve the accuracy of differential gene expression analysis (Soneson et al. 2015).

The complete RNA-seq dataset that was generated in this study consisted of 16 samples (8 treatments × 2 replicates), including also samples from T47D cells exposed to a series of retinoids, including all-*trans*-retinoic acid (AtRA) (Sigma), and the synthetic retinoids 4-[(5,6,7,8-tetrahydro-5,5,8,8-tetramethyl-2-naphthalenyl)carbamoyl] benzoic acid (Am80)(Abcam, Cambridge, UK), 5-(5,6,7,8-tetrahydro-5,5,8,8-tetramethyl-2-anthracenyl)-3-thiophenecarboxylic-acid (CD2314)(Tocris Bioscience, Bristol, UK) and 3-fluoro-4-[[2-hydroxy-2-(5,5,8,8-tetramethyl-5,6,7,8,-tetrahydro-2-naphthalenyl) acetyl]amino]-benzoic acid (BMS961)(Tocris Bioscience). Although not all treatments are of relevance to address the research question posed in this paper, all samples were included in the statistical analyses. This was done because this improves the empirical Bayes gene-wise dispersion (variability) estimates, which is advantageous when having two replicates per group (see below).

Before statistical analyses, nonspecific filtering of the count table was performed to increase detection power (Bourgon et al. 2010) based on the requirement that a gene should have an expression level greater than 10 counts, i.e. 0.65 count per million reads (cpm) mapped, for at least 2 libraries across all 16 samples. Differences in library size were adjusted by the trimmed mean of M-values normalization method (Robinson and Oshlack 2010). Differentially expressed genes were identified by using generalized linear models that incorporate empirical Bayes methods that permit the estimation of gene-specific biological variation, thereby improving testing power (Lun et al. 2016; McCarthy et al. 2012; Robinson and Smyth 2007). When indicated, thresholded hypotheses testing using a log2 fold-change of 0.6 was performed to identify robustly regulated genes, and genes regulated by a fold-change below this threshold were considered not to be biologically meaningful (McCarthy and Smyth 2009). In all cases, genes that satisfied the criterion of moderated *p* value < 0.05 were considered to be significantly regulated. For the general overview, as shown in the heatmap (Fig. 6a), only the criterion of false discovery rate (FDR) < 0.05 (Benjamini and Hochberg 1995) in any of the 3 comparison was considered to select significantly regulated genes.

Gene ontology and pathway analysis were carried out using the Consensus Path Database (cpdb) tool (Kamburov et al. 2011). For NR pathway analysis, lists containing all cpdb and the top 100 genes of the NURSA data base Transcriptomine (Consensome) (Becnel et al. 2015) were also used. In all cases, only gene ontology and pathways with *p* values lower than 0.05 were included for analysis. In addition, other web tools such as Heatmapper (Babicki et al. 2016) and Interactivenn (Heberle et al. 2015) were used to create the heatmaps and Venn diagrams, respectively.

### Gene expression (RT-qPCR) studies

Real-time quantitative polymerase chain reaction (RT-qPCR) amplification reactions were carried out to confirm the genes that showed significant and biologically relevant expression in the RNA-seq analysis. To this end, T47D or MCF-7 cells were seeded in 25-cm^2^ flasks (Corning, NY, USA) using growth medium, which, after the cells reached 50–60% confluence, was replaced by phenol red free medium. Twenty-four hours later, cells were exposed to the test compounds in phenol red free medium for 6 h. Following the exposure, cell lysis was carried out using RLT Lysis buffer (Qiagen, Venlo, The Netherlands). Total RNA was extracted using QIAshredder and RNeasy kits (Qiagen, Venlo, The Netherlands) according to the manufacturer’s instructions. Spectrophotometric analysis was performed using a Nanodrop (ND-1000 Thermoscientific Wilmington, Delaware, USA) to quantify and ensure the quality of the RNA. Next, RNA was converted into cDNA using the QuantiTect Reverse Transcription Kit (Qiagen, Venlo, The Netherlands). Expression of GAPDH (housekeeping gene) and HDAC7, HDAC11, HIST1H2BE, CPP26A1, CYP26B1, TFF1, AXIN2 and CXCL12 were quantified by RT-qPCR using Rotor-Gene SYBR® Green Kit (Qiagen, Venlo, The Netherlands) and the Rotor-Gene 6000 cycler (Qiagen, Venlo, The Netherlands) following the manufacturer’s protocol. To do so, this study made use of the QuantiTect Primer Assays Hs_GAPDH_1_SG, Hs_HDAC7_1_SG, Hs_HDAC11_1_SG, Hs_HIST1H2BE_1_SG, Hs_CYP26A1_1_SG and Hs_ CYP26B1_1_SG, Hs_TFF1_1_SG, Hs_AXIN2_1_SG and Hs_CXCL12_1_SG (Qiagen, Venlo, The Netherlands).

## Results

### Activation of ERα-mediated gene expression in the U2OS-ERα luciferase reporter gene assay and induction of T47D cell proliferation

**Fig. 2:**
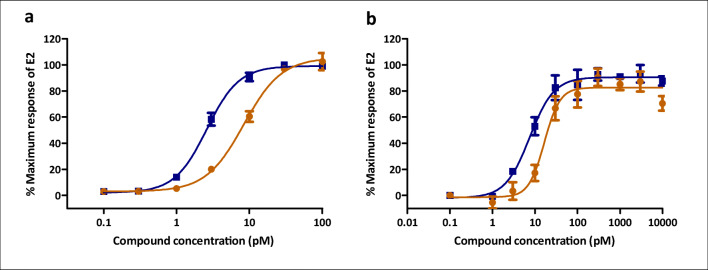
Concentration dependent ERα-mediated induction of (**a**) luciferase activity in U2OS-ERα reporter gene cells by E2 (blue) and DES (orange) and of (**b**) T47D cell proliferation after 72 h of exposure to E2 (blue) and DES (orange). Each data point represents the mean of three independent experiments ± SD

Treatment of human U2OS-ERα luciferase cells with DES and E2 resulted in concentration-dependent induction of luciferase expression (Fig. 2a). Induction of ERα-mediated luciferase expression by DES and E2 occurs at concentrations between 1 and 100 pM in a similar manner. From the results obtained, the EC50 values for DES and E2 were determined (Table 1). The EC50 value is 3-fold lower for E2 compared with that of DES, indicating a higher potency of E2 for induction of ERα-mediated gene expression. In subsequent experiments, the DES- and E2-induced ERα-mediated proliferation of T47D human breast cancer cells was investigated. After 72 h of exposure, both DES and E2 increased T47D cell proliferation in a concentration-dependent manner (Fig. 2b). The EC50 values derived from these curves were 2-fold lower for E2 than those for DES as presented in Table 1.

**Table 1:** EC50 values (95% confidence intervals) (pM) of DES and E2 as derived from the data presented in Fig. 2

Assay	EC50 E2 (pM)	EC50 DES (pM)
U2OS-ERα reporter gene expression	2.5 (2.3–2.9)	8.4 (7–9.9)
T47D cell proliferation	7.5 (5.3–10.6)	16.6 (11.9–23.3)

### ERα-mediated coregulator motif binding induced by DES and E2

**Fig. 3:**
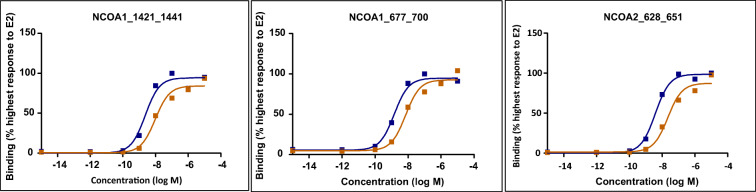
E2 (blue) and DES (orange) concentration-dependent induction of ERα-LBD binding to coregulator motifs (NCOA1_1421_1441, NCOA1_677_700 and NCOA2_628_651)

The ligand-induced interaction of the ligand binding domain of ERα (ERα-LBD) with coregulator motifs was characterized in the MARCoNI coregulator binding assay, in order to evaluate and compare the capacity of DES and E2 to modulate ERα-LBD binding to coregulator motifs. Most of the coregulator motifs showed an increased binding signal with increasing DES and E2 concentration (Supplementary material 1). As an example, Fig. 3 presents the concentration-dependent induction by DES and E2 of the interaction of ERα-LBD with NCOA1_1421_1441, NCOA1_677_700 and NCOA2_628_651. The observation of an increase in binding with increasing concentration of the model compounds is in line with the role of these compounds as receptor agonist and the function of these three coregulators as coactivators. The results reveal a similar concentration-dependent induction of ERα-mediated coregulator motif binding for these three coregulators with the EC50 for E2 being about 1.5-fold lower than that of DES.

### Comparison of the effects of DES and E2 on coregulator motif binding to ERα

**Fig. 4:**
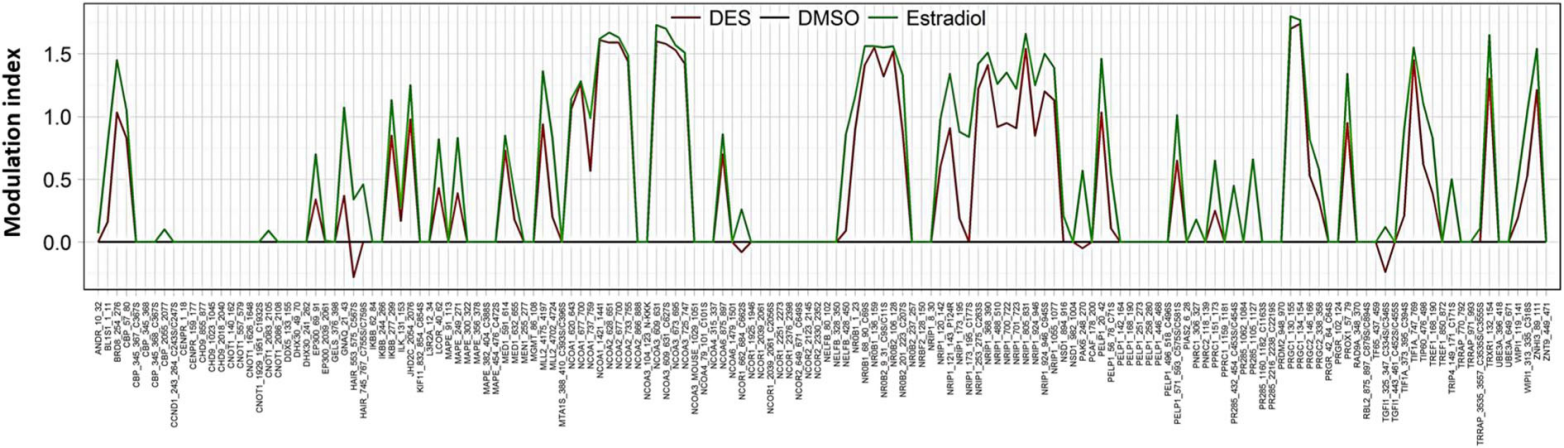
E2 (green) and DES (red) cause similar ERα-LBD coregulator binding patterns. The MI represents the ligand-induced modulation of ERα-LBD binding to coregulator motifs by DES and E2 both tested at 10^−6^ M, compared with the solvent control

The concentration-response data obtained for all 154 coregulator motifs present on the array show that, for both E2 and DES, maximum responses were obtained at 10^−6^ M (Fig. 4; supplementary material 1). To compare DES-induced and E2-induced ERα–coregulator interactions, the modulation index (MI) profile was determined (Fig. 4), defined as the log fold modulation of ligand-induced ERα-LBD-mediated binding with different coregulator motifs in the presence of 10^−6^ M DES or E2 compared with the solvent control. In this MI profile the changes in ERα-LBD binding to the coregulator motifs are expressed relative to the solvent control (DMSO). Positive values on the y-axis present higher binding than the solvent control and negative values reflect lower binding. Binding patterns induced by DES and E2 appear to be overall quite similar, with the major difference being that for DES the MI values for a large number of coregulator motifs are lower than for E2 (Fig. 4). In the next step, these differences were analyzed to a further extent.

**Fig. 5:**
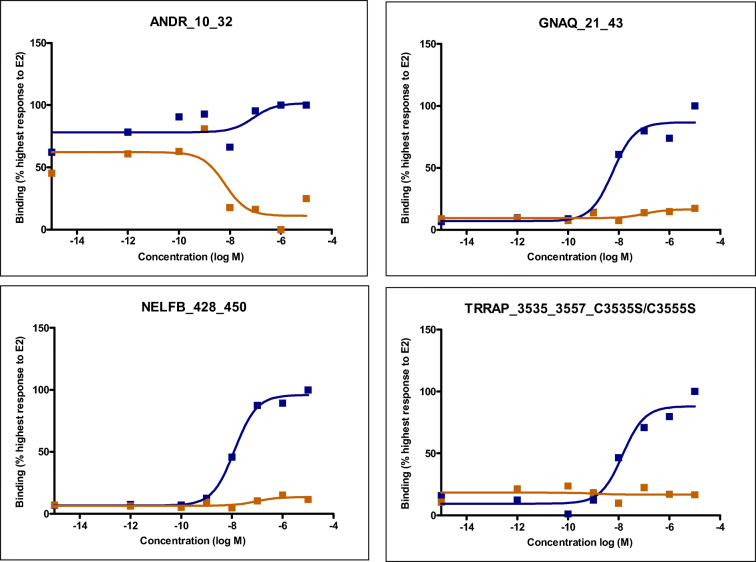
E2 (blue) and DES (orange) concentration-dependent induction of ERα-LBD binding to coregulator motifs ANDR_10_32, GNAQ_21_43, NELFB_428_450 and TRRAP_3535_3557_C3535S/C3535S that show differences between E2 and DES

To further investigate to what extent coregulator binding may differentiate the ERα agonist action of DES and E2, the coregulator motifs that show concentration-response curves with a coefficient of determination (R^2^) ≥ 0.8 for at least either E2 or DES were selected for further analysis. All the concentration-response curves with R^2^ ≥ 0.8 are presented in supplementary material 1 with the response expressed as percentage of the highest response to E2 for the respective coregulator motif set at 100% and the effect of the solvent control at 0%. This analysis reveals that 78 out of 154 coregulators motifs gave adequate concentration response curves with R^2^ ≥ of 0.8 for E2 and/or DES. From these 78, 63 coregulators motifs gave adequate concentration-response curves for both DES and E2, while 14 show a response only for E2 and one only for DES. Concentration-response curves for 4 of the 15 coregulator motifs that show a differential response toward DES and E2 are presented in Fig. 5, while the concentrations-response curves for the other coregulators motifs are presented in supplementary material 1.

Table 2 presents these 15 coregulator motifs and the biological function of the corresponding coregulator as far as these are known. The 15 coregulator motifs belong to 11 coregulators. The function of several of the coregulators of which a respective motif interacts specifically with E2 only are coactivators that enhance gene transcription (CBP, MLL2, NRIP1, TIF1A, TRIP4 and TRRAP) while others act as co-repressors (NCOR1, NELFB, NRIP1 and PAK6). Also of interest to note is that several of the coregulators of which a respective motif specifically responds to E2 and not to DES influence histone (de)acetylation. One coregulator motif, ANDR_10_32, responded only to DES showing a decrease in ERα-LBD binding with increasing concentration of DES that was not observed with E2 as presented in Fig. 5. The function of the corresponding coregulator ANDR of which motif ANDR_10_32 shows a DES-specific response is not known. However, given the decrease in binding upon DES interaction with the ERα-LBD and the fact that it is an androgen receptor–related coregulator suggest it may be an estrogen receptor co-repressor, resulting in activation of estrogen-related gene transcription upon its DES-induced release. To what extent such subtle differences in coregulator interactions might result in differences in gene transcription was investigated using next generation sequencing (RNA-seq) and transcriptome analysis.

**Table 2:** Overview of the 15 coregulator motifs that show a differential agonist-induced ERα-dependent binding response for DES and E2

Coregulator motif	DES	E2	Coregulator name/family	Function
ANDR_10_32	+	−	Androgen receptor–related coregulator	Unknown
CBP_2055_2077	−	+	CREB-binding protein	Coactivator for nuclear receptors (NRs) enhancing histone acetylation (Hung et al. 2001; Vincek et al. 2018)
GNAQ_21_43	−	+	Guanine nucleotide-binding protein	Unknown
MLL2_4702_4724	−	+	Myeloid/lymphoid or mixed-lineage leukemia protein 2	Part of a complex that acts as coactivator for estrogen receptor alpha and shown to be a transcriptional regulator of β-globin (Demers et al. 2007; Mo et al. 2006). MLL2 is also implicated in the regulation of methylation of histone 3 at lysine 4 (H3K4) (Zhao et al. 2016).
NCOR1_662_684_C662S	−	+	Nuclear receptor corepressor 1	NCOR1 mediates transcriptional repression by different nuclear receptors. It is part of a complex which promotes histone deacetylation and the formation of repressive chromatin structures (Cui et al. 2011; Yoon et al. 2003).
NELFB_428_450	−	+	Negative elongation factor B	NELFB in complex negatively regulates transcription elongation and causes transcriptional repression (Narita et al. 2003; Yamaguchi et al. 1999).
NELFB_80_102	−	+		
NRIP1_173_195	−	+	Nuclear receptor-interacting protein1	NRIP1 can both co-activate and corepress transcription mediated by nuclear receptors including ERs (Castet et al. 2004; Cavailles et al. 1995; Subramaniam et al. 1999).
NRIP1_173_195_C177S	−	+		
PAK6_248_270	−	+	Serine/threonine-protein kinase PAK6	PAK6 kinase plays a role in the regulation of gene transcription. It is reported to inhibit androgen receptor and ERα-mediated transcription by phosphorylation of the DNA binding domain (Lee et al. 2002; Zhang et al. 2010).
PR285_2216_2238_C2219S	−	+	Peroxisomal proliferator-activated receptor A-interacting complex 285 kDa protein PRIC285: PPAR-alpha-interacting complex protein 285	Unknown
PR285_432_454_C453S/C454S	−	+		
TIF1A_373_395_C394S	−	+	Transcription intermediary factor 1-alpha TRIM24: tripartite motif containing 24	TIF1A is a transcriptional coactivator that interacts with numerous nuclear receptors and coactivators and modulates the transcription of target genes. Furthermore, it is reported to play a role in regulation of cell proliferation and apoptosis by regulating p53 level (Allton et al. 2009; Thenot et al. 1997).
TRIP4_149_171_C171S	−	+	Thyroid receptor-interacting protein 4	Acts as a transcriptional coactivator and plays a role in different transactivation of nuclear receptors including ERs and thyroid hormone receptors (Kim et al. 1999; Yoo et al. 2014).
TRRAP_3535_3557_C3535S/C3555S	−	+	Transformation/transcription domain-associated protein	Coactivator TRRAP is an adapter protein complex that induces epigenetic transcription activation by histone acetyltransferase activity. It also plays a role in transcription activation of proto-oncogene MYC and tumor suppressor genes p53 (Ard et al. 2002; Lang and Hearing 2003; Liu et al. 2003; McMahon et al. 1998).

### Transcriptome analysis of T47D cells exposed to DES and E2

**Fig. 6:**
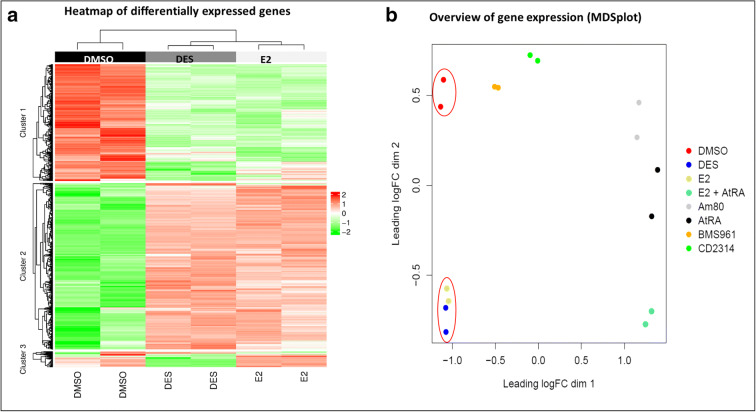
General overview of the RNA-seq assessment for T47D cells exposed to E2 or DES (10 nM) compared with the solvent control (DMSO) presented in (**a**) a heatmap of differentially expressed genes significantly different (FDR < 0.05) in at least 1 of the treatments (red, high expressed genes; green, low expressed genes); and (**b**) Principal Coordinates Analysis plot for E2, DES and the solvent control (DMSO) also including—to facilitate comparison—the data for 6 other treatment groups analyzed in the same experiment, including all-trans retinoic acid (AtRA) and the synthetic retinoids 4-[(5,6,7,8-tetrahydro-5,5,8,8-tetramethyl-2-naphthalenyl)carbamoyl] benzoic acid (Am80), 5-(5,6,7,8-tetrahydro-5,5,8,8-tetramethyl-2-anthracenyl)-3-thiophenecarboxylic-acid (CD2314) and 3-fluoro-4-[[2-hydroxy-2-(5,5,8,8-tetramethyl-5,6,7,8,-tetrahydro-2-naphthalenyl) acetyl]amino]-benzoic acid (BMS961)

In the next step, the effects of DES and E2 on the gene expression in T47D cells was characterized using transcriptome analysis (RNA-seq) to better understand the potential differences between DES- and E2-induced ERα activation. An overview of the RNA-seq analysis of T47D cells exposed to DES and E2 is presented in Fig. 6. The heatmap (Fig. 6a) provides a visual representation of the differences in gene expression between DES and E2 and the solvent control (DMSO). The results of a Principal Coordinates Analysis presented in Fig. 6b also include the data from a series of retinoids tested in the same experiment thus showing clearly that DES- and E2-induced differential modulation of gene expression is different from that of the solvent control and also from the retinoids tested at the same time, while the differences between DES and E2 appear to be relatively small, albeit consistent.

**Fig. 7:**
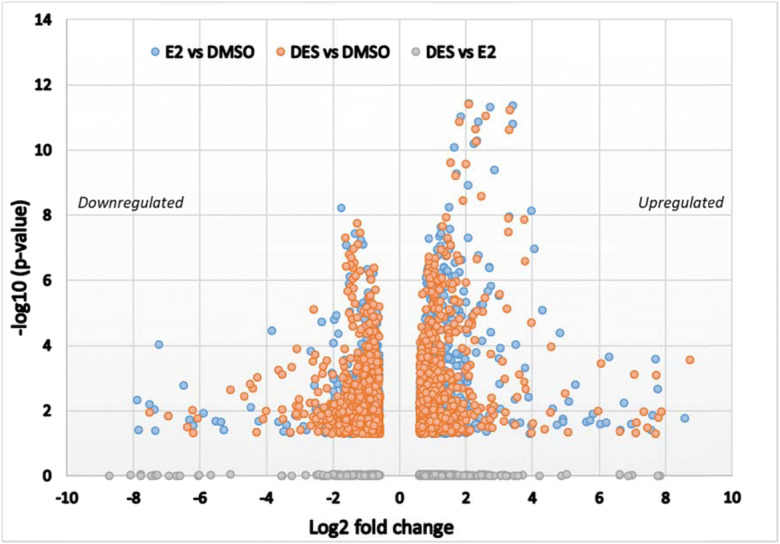
A volcano plot showing differential expressed genes (upregulated and downregulated). In the figure, each dot represents a gene showing the log2 fold-change and the -log10 (moderated *p* value). Genes with significant expression changes (compared with DMSO) have a large magnitude fold change and high statistical significance (low *p* value) The genes included in volcano plot are those with log2 FC ≥ 0.6 and moderated *p* value < 0.05

Figure 7 shows the volcano plot presenting the total number of up- and downregulated genes thus obtained and their overlap between DES and E2. The results obtained reveal that the total number of genes upregulated by E2 and DES are higher than the downregulated genes.

### Gene ontology and pathway analysis

To gain insight into the biological meaning of the gene expression data, first a gene ontology overrepresentation analysis was performed using the consensus path database tool. For the gene ontology, the total number of genes regulated by DES and E2 compared with DMSO is presented based on their moderated *p* value and their gene ratio to the total number of genes that are involved in a specific biological process. The overview of all biological process categories for the overrepresented genes by either DES versus DMSO or E2 versus DMSO is presented in supplementary material 2. This overview reveals that, in spite of the relatively large number of DEGs specific for DES and E2 alone, DES and E2 show similar patterns with only small differences like those for the GO category *gland development* (only overrepresented in DES/E2 treatment) and *response to the retinoid receptor* (only overrepresented in DES/E2 treatment).

**Fig. 8:**
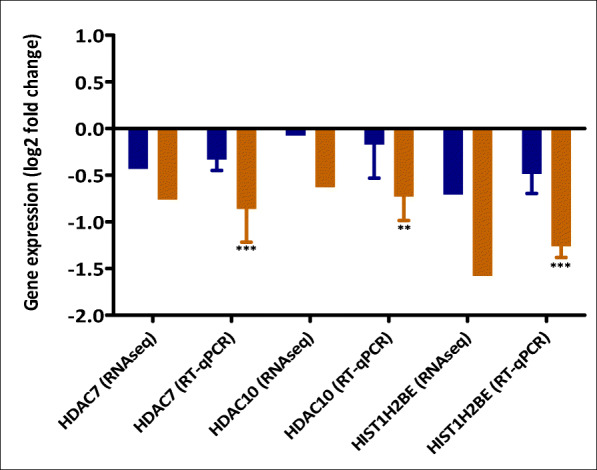
Example of genes that are significantly downregulated by DES (orange) only that are involved in histone deacetylation and related DNA methylation. The expression was considered significant if log2 FC > 0.6 and moderated *p* value < 0.05. For RT-qPCR results, bars represent average ± SEM from at least three independent experiments. For statistical analysis of the RT-qPCR data, multiple paired *t* tests were performed and differences were considered significant if *p* value < 0.05

Next, a pathway overrepresentation analysis, using the consensus path database tool and the NURSA database, was performed for the three groups of genes, E2- and DES-induced DEGs and DEGs induced by either DES or E2 alone. Supplementary materials 3, 4 and 5 present the pathways analyses for these 3 DEG categories. It is clear from the pathway analysis of DEGs induced by both E2 and DES (supplementary material 3), that DES and E2 regulate pathways related to ERα network significantly with a very low *p* value. Furthermore, DES and E2 shared multiple pathways like *mammary gland development*, *breast cancer* and *the estrogen receptor pathway*. DEGs of interest that were specifically regulated by DES (supplementary material 4) appeared to relate especially to genes that relate to possible epigenetic effects, such as the relatively high level of downregulation of genes involved in histone modification and DNA methylation. Differential expression of three genes upon exposure of the cells to especially DES was confirmed by RT-qPCR. Figure 8 presents the results obtained corroborating the significant downregulation of the expression of genes involved in histone deacetylation (like HDAC10 and HDAC7) and DNA methylation (HIST1H2BE) upon exposure to DES but not E2. Figure 8 reveals that DES (grey) downregulated these genes significantly compared with E2 (black). The differential expression of HDAC10, HDAC7 and DNA HIST1H2BE were validated using qPCR in T47D, and, moreover, the differential expression of the HDAC10 and HDAC7 genes were also validated in the MCF-7 cell line (the data are presented in the supplementary material 7).

Pathway analysis for the genes that were regulated by E2 only (Supplementary material 5) revealed that most of these pathways relate to transforming growth factor (TGF)-related pathways such as the *BMP signalling* pathway, *BMP2 signalling TGF-beta MV*, *BMP signalling Dro* and *BMP receptor signalling.*

### Analysis of differential gene expression in nuclear receptor pathways involved in developmental processes and toxicity

**Fig. 9:**
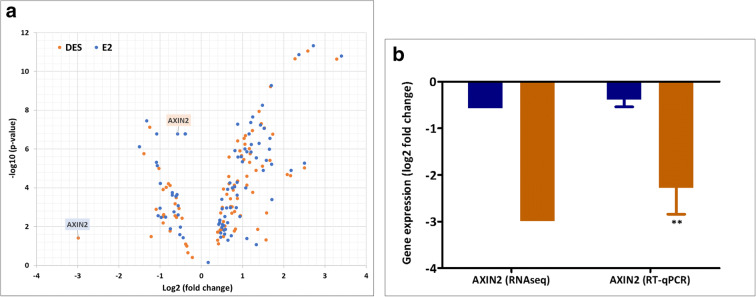
RNA-seq and RT-qPCR characterization of the effects of DES and E2 on gene expression associated with the ER pathway. **a** displays a volcano plot showing all genes related to ER signalling presenting significant changes induced by at least one of the two compounds (log2 FC ≥ 0.6 and moderated *p* value < 0.05). **b** presents RT-qPCR data for AXIN2, an ER-mediated gene that showed large differences between DES (blue) and E2 (green). For the volcano plot, each dot represents a gene showing the log2 fold-change and the -log10 (moderated *p* value). For RT-qPCR, results are expressed as log2 fold changes in relation to the solvent control. For RT-qPCR results, bars represent average ± SEM from at least three independent experiments. For statistical analysis of the RT-qPCR data, multiple paired *t* tests were performed and differences were considered significant if *p* value < 0.05

To further elucidate gene expression results that may explain the differential developmental toxicity of DES and E2, pathways that relate to ER, retinoid acid receptor (RAR) and estrogen-related receptor (ERR) related nuclear receptor signalling were analyzed in more detail. First, the differential effects on genes related to ER signalling were evaluated (Fig. 9). Figure 9 a displays the log2 fold changes induced by DES and E2 for the transcription of genes known to play a role in ER-mediated pathways. In addition, Fig. 9b shows RT-qPCR data focusing on selected ER-mediated gene that significant differences between DES and E2. As shown in the volcano plot and the bar graphs, almost all the genes that were differentially regulated by DES and E2 were regulated in a similar way by the two ER agonists. However, DES specifically downregulated the E2-responsive gene AXIN2, an effect not observed upon E2 exposure. The expression of this gene is also validated in MCF-7 cells, showing also effect by DES not observed for E2, and the results are presented in the supplementary material 7.

**Fig. 10:**
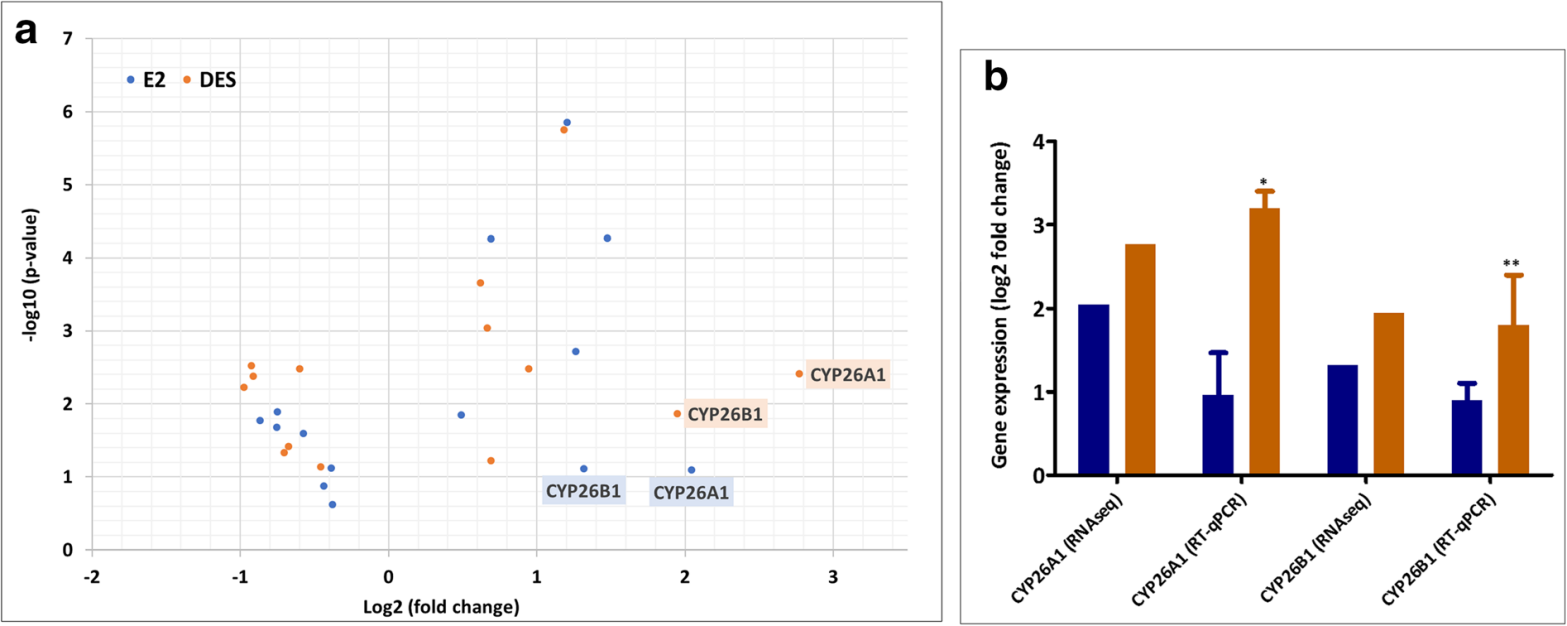
RNA-seq and RT-qPCR characterization of the effects of DES and E2 on gene expression associated with RAR pathways. **a** displays a volcano plot showing all genes related to RAR signalling presenting significant changes induced by at least one of the two compounds**. b** presents RT-qPCR data for CYP26A1and CYP26B1 in RAR-mediated gene that showed high differences between DES (orange) and E2 (blue). For the volcano plot, each dot represents a gene showing the log2 fold-change and the -log10 (moderated *p* value). For RT-qPCR, results are expressed as log2 fold changes in relation to the solvent control. For RT-qPCR, results are expressed as log2 fold changes in relation to the solvent control. The change in expression is considered significant if log2 FC > 0.6 and the *p* value < 0.05. For RT-qPCR results, bars represent average ± SEM from at least three independent experiments. For statistical analysis of the RT-qPCR data, multiple paired *t* tests were performed and differences were considered significant if *p* value < 0.05

Considering the important role of the retinoid receptors in developmental processes and toxicity (Kam et al. 2012; Mark et al. 2009; Rhinn and Dolle 2012), gene expression associated with the retinoid acid receptor (RAR) pathway was also analyzed in more detail. Figure 10 a displays the fold changes obtained upon exposure of T47D cells to DES and E2 for the transcription of genes known to play important roles in the RAR pathway based on the pathway database. In addition, Fig. 10b presents RT-qPCR data focusing on selected RAR-mediated genes of which the expression was affected to a large extent by E2 and/or DES. Most RAR-dependent genes are regulated by E2 and/or DES in a similar manner. However, DES significantly upregulated CYP26A1 and CYP26B1 expression, an effect not observed at a significant level upon exposure of the T47D cells to E2 (Fig. 10b). These genes were also validated in MCF-7 cells, showing also an effect by DES not observed for E2, and the data are presented in the supplementary material 7.

Finally, possible differential expression of ERRs pathways was evaluated in more detail. DES is known to interact with ERRs (Nam et al. 2003), while E2 is reported to not interact with ERRs. Supplementary material 6 presents the genes differentially affected by DES and E2 related to ERR signalling based on the pathway database. No significant differences between DES and E2 were found, and it was concluded that the expression of the genes involved in this pathway is very similar upon DES and E2 exposure.

In summary, the results from the ontology and pathway analysis and from the RT-qPCR data indicate that there are subtle albeit interesting and significant differences between DES and E2 in transcriptomic signatures obtained in the T47D cell line. Furthermore, these subtle differences were also observed in the MCF-7 cell line.

## Discussion

Adverse effects of DES have been reported to be mediated via the ERα (Couse et al. 2001; Couse and Korach 2004; Prins et al. 2001). This suggests that studying the molecular events related to ERα is crucial to understand the potential mode of action underlying DES-induced adverse effects. Given, however, that DES acts as an ERα agonist and thus via a mode of action potentially similar to the endogenous female hormone E2, it is of even more interest to elucidate the potential differences between DES- and E2-induced ERα-mediated effects. The objective of this study was to assess whether DES and E2 differ in their ERα-mediated responses, aiming to provide information on possible underlying differences in their mode of action and resulting potential developmental toxicity. To this end, the activities of DES and E2 were compared in a series of ERα-related bioassays including the U2OS ERα reporter gene assay, T47D cell proliferation assay, ligand-induced ERα-mediated coregulator interaction and gene expression profile in ERα positive T47D cells as well as ERα positive MCF-7 cells.

DES and E2 acted as ERα agonists in the U2OS-ERα cells (Fig. 2a) and increased T47D proliferation (Fig. 2b) in a similar manner, with the potency (reflected by the EC50) of E2 being only slightly higher than that of DES (Table 1). These results are consistent with results from ERα reporter gene and cell proliferation data for ERα positive cells reported in the literature (Kalach et al. 2005; Sotoca et al. 2008). However, the binding affinity of DES to ERα has been reported to be slightly greater than that of E2 (Blair et al. 2000; Bolger et al. 1998; Okulicz and Johnson 1987; Shelby et al. 1996).

Transcriptional activation mediated via the ER and other nuclear receptors is influenced by binding to transcriptional coregulators that can activate (eg. NCOA) or repress (NCOR) the gene transcription (Glass and Rosenfeld 2000; O'Malley and Kumar 2009). Previous research showed that overexpression or lack of certain ligand-dependent coregulators could affect the physiological outcome driven by a chemical (Hsia et al. 2010). Therefore, the interaction of the ERα with coregulators in the presence of DES and E2 was studied to obtain further insight in possible differences in their modes of action. Results obtained revealed that DES and E2 displayed similar responses with only a few, albeit significant, differences in the ligand-induced coregulator motif binding pattern to the ERα-LBD. A high number of coactivator and corepressor motifs were found to interact with ERα in a DES and E2 concentration-dependent manner, suggesting that a broad range of coregulator proteins is involved in ERα signalling induced by both agonists. However, 15 out of 154 coregulators motifs showed a marked difference in their response to DES and E2. These 15 coregulator motifs appear to belong to 11 coregulators including ANDR, CBP, GNAQ, MLL2, NCOR1, NELFB, NRIP1, PAK6, PR285, TRIP4 and TRRAP. Of these coregulator motifs, only ANDR_10_32 showed a DES-specific response, while the other 14 bound to the ERα-LBD in the presence of E2 and not in the presence of DES (Fig. 5). Interestingly, in the presence of DES, the ERα-LBD bound to other coregulator motifs on the chip of CBP, MLL2, NCOR1, NRIP1 and PR285 than the coregulator motifs of the coregulators presented in Table 2 that specifically interacted with E2 alone. Together, the data suggest a possible difference in ERα-coregulator interaction between DES and E2. For the coregulator motifs present on the chip of the coregulators GNAQ, NELFB, PAK6, TRIP4 and TRRAP, binding to the ERα-LBD was only observed in the presence of E2 and not in the presence of DES, thus pointing at additional specific differences in coregulator binding upon binding of DES or E2 to the ERα-LBD that have not been described in literature before. It is of interest to consider the role of these coregulators, although not all of them have been studied in detail so far.

The coregulator motif ANDR_10_32 responded only to DES, showing a decrease in ERα-LBD binding with increasing concentration of DES that was not observed with E2. The function of the corresponding androgen receptor–related coregulator (ANDR) is not known, but the loss of the interaction of this coregulator with ERα in the presence of DES, but not E2, might play a role in the reproductive tract effects of DES since it has been reported that the androgen receptor plays a role in mediating DES-induced effects in prostatic enlargement (Gupta 2000).

Another important finding was that binding of E2, but not of DES, to the ERα induced binding of motifs of the corepressors NELFB and PAK. Both PAK and NELFB are considered corepressors for ERα function reducing its transcriptional activities (Aiyar et al. 2004; Lee et al. 2002). Furthermore, a lack of NELFB expression in breast carcinoma may serve as a useful indicator for poor prognosis (Aiyar et al. 2007; Sun et al. 2008), thus pointing at a beneficial role for NELFB. The recruitment of the coregulator TRRA upon binding of E2 to ERα is consistent with the literature. It has been reported that E2 induces direct binding of ERα to TRRAP (Fujita et al. 2003). TRRAP has been reported to play different roles in cell cycle and histone transcription (DeRan et al. 2008; Ichim et al. 2014). The difference in recruitment of TRRAP by E2 and not by DES may thus contribute to the differential biological responses induced by the two ERα agonists. Other coregulator motifs and related coregulators that appeared to respond different to E2 and DES have not been studied in detail, so a clear role in the differential biological responses to DES and E2 is less obvious.

To further assess subtle differences in cellular responses induced by DES and E2, gene expression in DES- and E2-exposed ERα competent T47D cells were assessed using RNA seq. An initial view and Principal Coordinates Analysis of the general transcriptomes induced by the test compounds showed that DES and E2 clustered together and were clearly grouped apart from a series of retinoids, also known to cause developmental toxicity, tested in the same experiment (Fig. 5b). General comparison of the heatmaps confirmed that DES and E2 presented remarkably similar expression patterns and levels although close analysis of the data revealed minor, albeit significant differences as shown in the heatmap (Fig. 6a).

The biological consequences of the genes that show specific regulation by either DES alone or E2 alone or genes that were regulated by both estrogens were evaluated in a subsequent pathway analysis. Interestingly, pathway analysis for the genes regulated specifically by DES highlighted potential differential epigenetic effects induced by DES compared with E2, including effects on genes involved in histone modification and DNA methylation. Histone deacetylase–related genes HDAC7, HDAC10 and HISTIH2BE were significantly downregulated by DES while not by E2 (Fig. 8). These findings are consistent with previous research that reported DES-induced histone deacetylation in the promoter region of P450scc in TTE1 Leydig cells, while E2 did not induce these changes (Warita et al. 2010). Furthermore, DES exposure resulted in expression of certain genes (HIST1H3E, HIST1H3D, HIST1H2BE, HIST1H2BG and HIST2H2AA3) involved in DNA methylation pathways, while these genes did not show significant E2-induced regulation. This group of genes normally clusters together and is highly expressed during the S-phase of the cell cycle (Harris et al. 1991). It has been reported that aberrant DNA methylation was implicated in DES-induced reproductive developmental abnormalities and tumor formation (Newbold et al. 2006; Sato et al. 2009). The differences observed in DES- and E2-mediated induction of genes involved in epigenetic modes of action, observed to a substantially higher extent for DES than for E2, can add to the observations that DES-mediated effects are transferred to subsequent generations via epigenetic modes of action (Doherty et al. 2010; Bromer et al. 2009).

Nuclear receptors act as ligand-inducible transcription factors by directly interacting with DNA response elements for the target genes. Therefore, nuclear receptor pathway analyses were performed to identify pathways potentially affected by E2 or/and DES through their interaction with ERs, RARs and ERRs since these nuclear receptors may play a role in modes of action underlying developmental toxicity (Collins and Mao 1999; Couse and Korach 2004; Luo et al. 1997; Willhite et al. 1996). Both compounds regulated multiple ER-related genes in a similar way (Fig. 9a). These ER-related genes were reported to play a role in ER-mediated regulation and can be target genes in breast cancer (Lin et al. 2004). From these estrogen-responsive genes, the AXIN2 gene appeared to be strongly downregulated specifically by DES as compared with E2 (Fig. 9b). This gene is reported to play a role in regulation of β-actin and inhibit the Wnt signalling pathway (Jho et al. 2002). The Wnt signalling pathway is essential for the embryonic developmental processes (Yang 2012), and the inhibition of this pathway by AXIN was associated with developmental toxicity and malformation in zebrafish (Heisenberg et al. 2001; Zhang et al. 2016).

Previous research has shown that DES can bind and activate estrogen-related receptors (ERRs) (Nam et al. 2003), while E2 cannot. These receptors share high homology to ERα (Eudy et al. 1998; Giguere et al. 1988) and regulate the activity of the estrogen-response element constitutively (Chen et al. 2001; Hong et al. 1999). Therefore, it was hypothesized that gene expression related to the ERR pathway might be influenced specifically by DES. However, the findings of the current study do not support this hypothesis. DES and E2 induced similar expression of ERR-related genes as presented in supplementary material 6. This high similarity between DES and E2 in ERR pathways might be due the fact that ERs and ERRs share high homology and might regulate many of the same genes (Vanacker et al. 1999a; Vanacker et al. 1999b).

The gene expression profile related to the RAR pathway was studied in more detail based on the fact that DES induces developmental toxicity in human and animals, a process in which retinoid acid signalling and timing of RAR activation play an important role (Cornwall et al. 1984; Nagao and Yoshimura 2009; Reed and Fenton 2013; Wardell et al. 1982). Furthermore, several agonists for the retinoid receptors like all-trans-retinoic acid and retinol have been found to induce developmental toxicity and to have a relation to breast cancer (Collins and Mao 1999; Garattini et al. 2014; Liu et al. 2015; Tembe et al. 1996; Turton et al. 1992). Therefore, the DES- and E2-mediated effects on RAR-mediated gene expression were also characterized in more detail. The analysis revealed that DES induced expression of especially CYP26A1 and CYP26AB1 to a significantly higher extent than E2 (Fig. 10b). These two genes are responsible for metabolism and elimination of retinoid acid (Loudig et al. 2000; Thatcher and Isoherranen 2009). DES-mediated upregulation of CYP26A1 and CYP26B1 gene expression is in line with the effects reported for the developmental toxins flusilazole and retinoic acid, which have been reported to increase the expression of these genes in a similar manner (Dimopoulou et al. 2016; Luijten et al. 2010). This effect may play an important role in the mode of action of DES in developmental toxicity.

Finally, it is important to note that concentrations used in the in vitro incubations were above physiological concentrations to be expected. However, the aim of the study was to detect potential mechanistic differences between DES- and E2-induced ERα-mediated cellular responses in order to create hypotheses for potential mechanistic differences between these ERα agonists. The extent to which these differences will be detectable in an in vivo setting remains to be investigated.

Altogether, it is concluded that the present study reveals further insight in possible modes of action underlying the differential biological effects of DES and E2. While effects of these two estrogens on ERα-mediated gene expression in an ERα reporter gene assay and on ERα-mediated cell proliferation were similar, coregulator binding and gene expression studies revealed subtle but significant differences. The studies on DES- and E2-induced coregulator binding to ERα-LBD showed differences for 15 coregulator motifs, and gene expression analysis revealed effects of DES on genes related to epigenetic regulation and developmental processes that were not observed for E2. These observations point at subtle differences in the estrogenic response that ultimately may contribute to their differential biological effects.

## Electronic supplementary material

ESM 1 (PDF 427 kb)

ESM 2 (PDF 7 kb)

ESM 3 (PDF 293 kb)

ESM 4 (PDF 247 kb)

ESM 5 (PDF 226 kb)

ESM 6 (PDF 42 kb)

ESM 7 (PDF 275 kb)
