# Supplementary material for: Estrogen receptor alpha (ERα)–mediated coregulator binding and gene expression discriminates the toxic ERα agonist diethylstilbestrol (DES) from the endogenous ERα agonist 17β-estradiol (E2)
Source: Cell Biol Toxicol. 2020 Feb 22;36(5):417–35. doi: 10.1007/s10565-020-09516-6 (PMC7505815; doi:10.1007/s10565-020-09516-6)
Supplement: Supplementary file 1 — (PDF 427 kb) [file 10565_2020_9516_MOESM1_ESM.pdf]

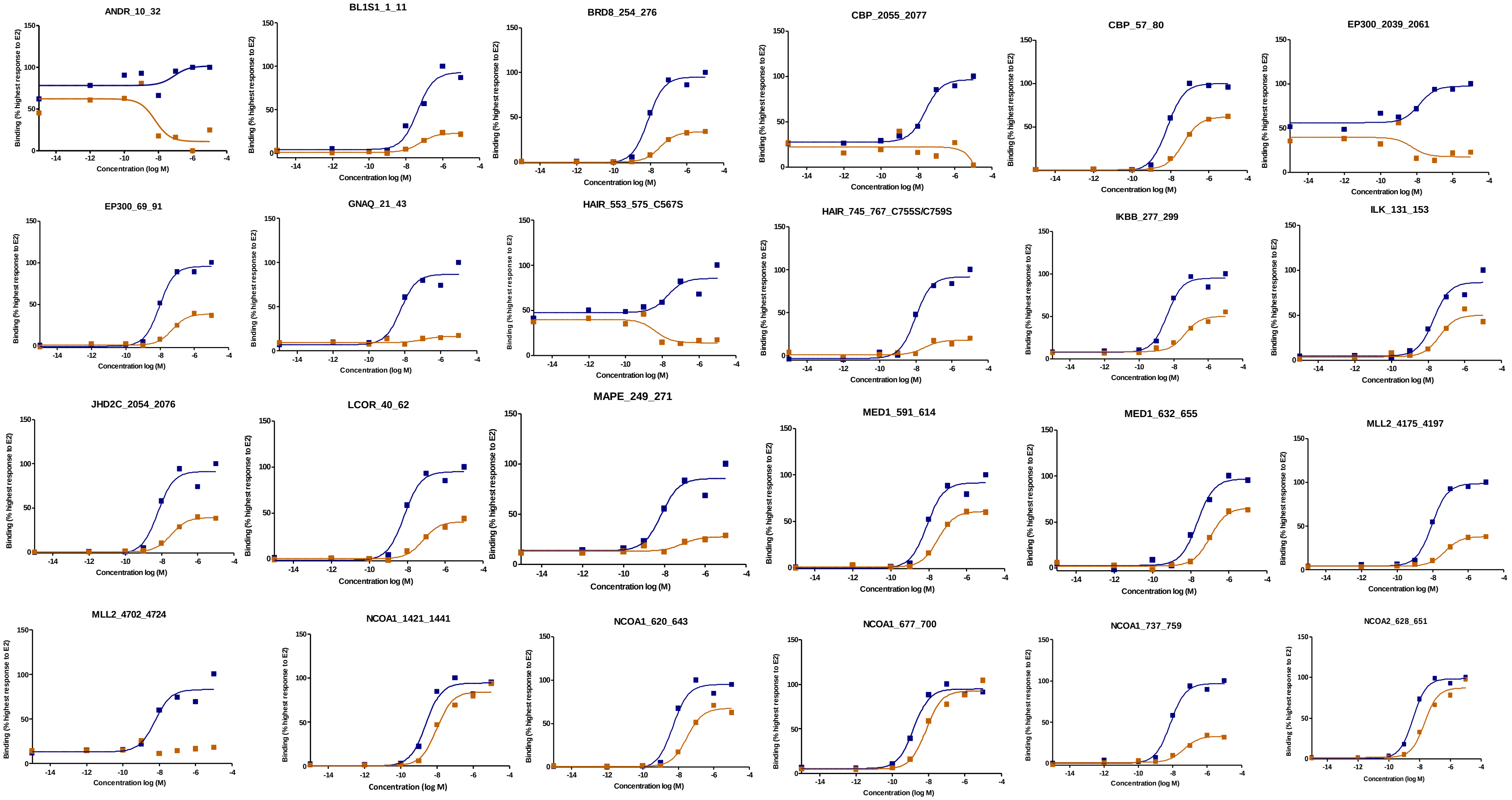

E2 (blue) and DES (orange) concentration dependent induction of ERα-LBD binding to coregulator motifs with coefficient of determination (R2) ≥ of 0.8 for at least either E2 or DES. The binding expressed as a percentage of maximum response of E2.

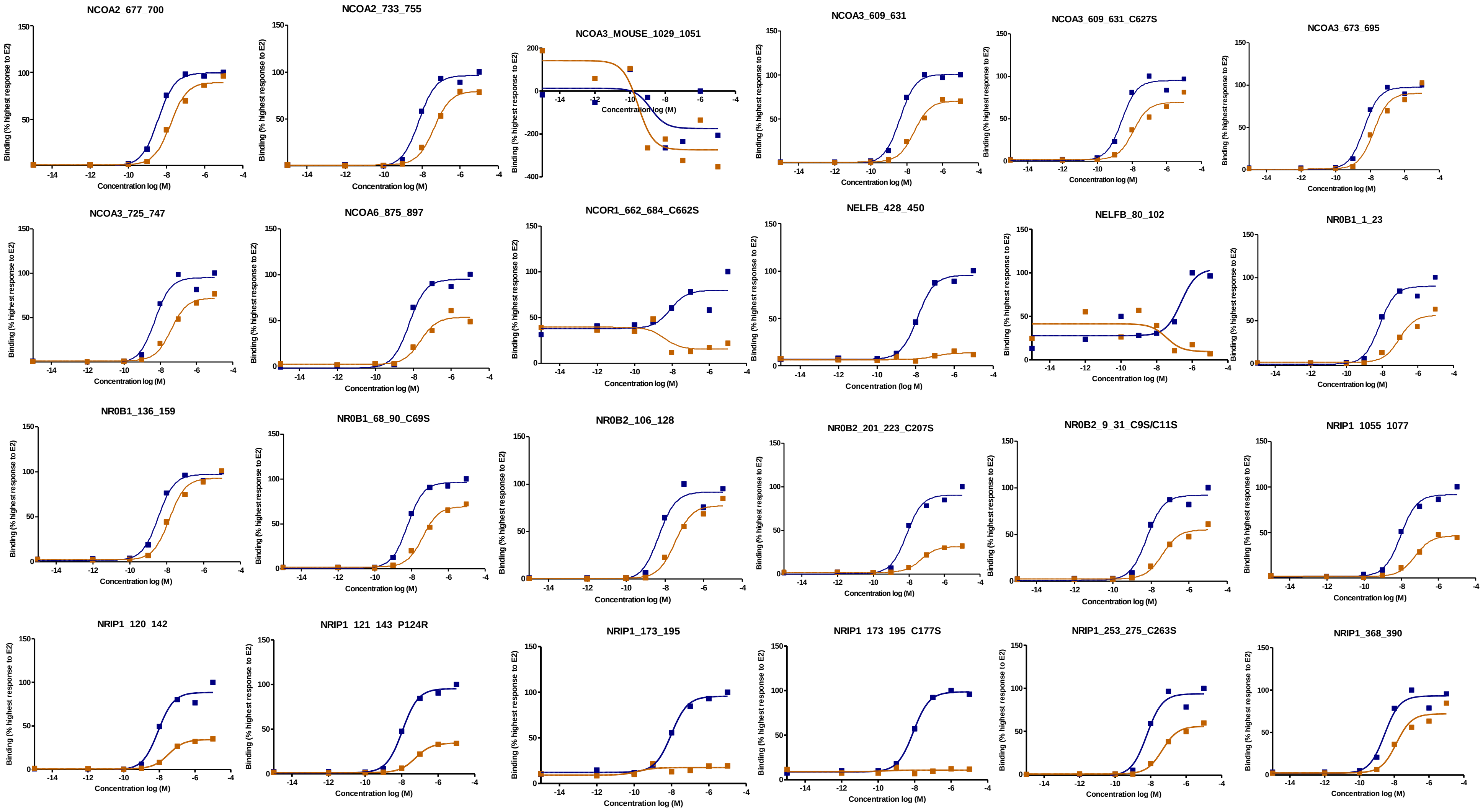

E2 (blue) and DES (orange) concentration dependent induction of ERα-LBD binding to coregulator motifs with coefficient of determination (R2) ≥ of 0.8 for at least either E2 or DES. The binding expressed as a percentage of maximum response of E2.

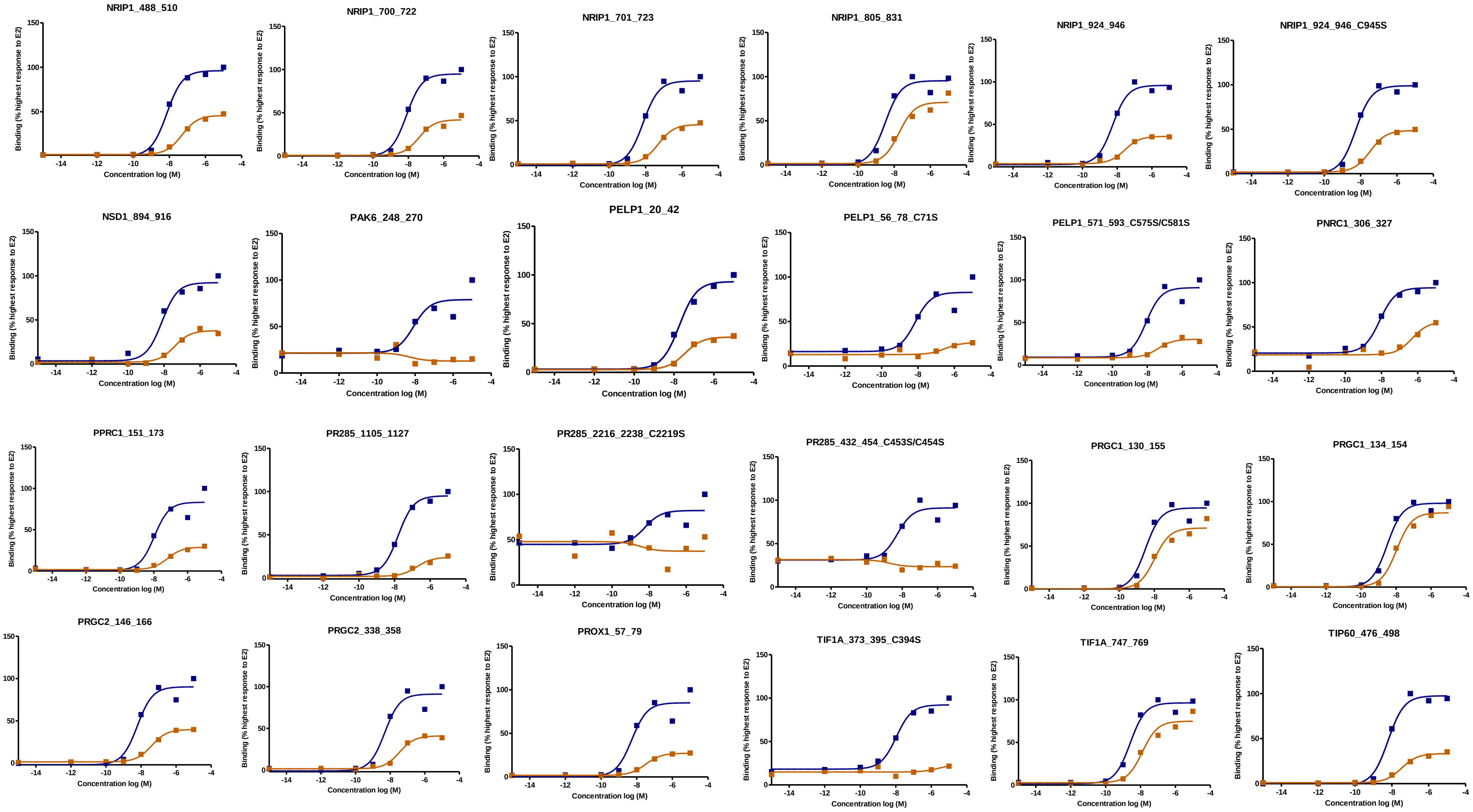

E2 (blue) and DES (orange) concentration dependent induction of ERα-LBD binding to coregulator motifs with coefficient of determination (R2) ≥ of 0.8 for at least either E2 or DES. The binding expressed as a percentage of maximum response of E2.

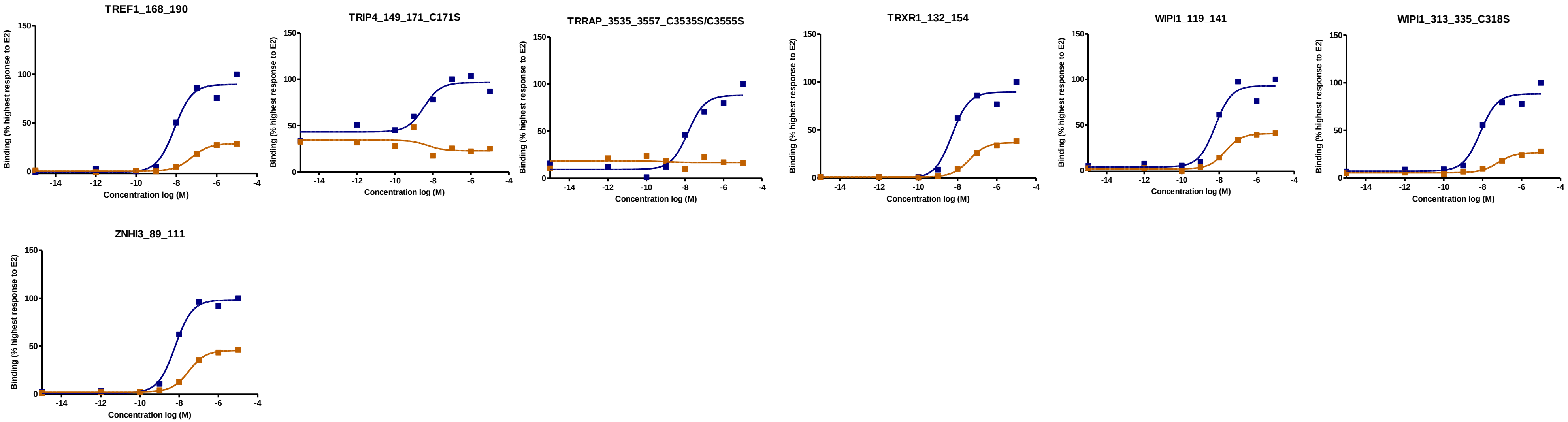

E2 (blue) and DES (orange) concentration dependent induction of ERα-LBD binding to coregulator motifs with coefficient of determination (R2) ≥ of 0.8 for at least either E2 or DES. The binding expressed as a percentage of maximum response of E2.
