# Supplementary figures and images for: Estrogen receptor alpha (ERα)–mediated coregulator binding and gene expression discriminates the toxic ERα agonist diethylstilbestrol (DES) from the endogenous ERα agonist 17β-estradiol (E2)
Source: Cell Biol Toxicol. 2020 Feb 22;36(5):417–35. doi: 10.1007/s10565-020-09516-6 (PMC7505815; doi:10.1007/s10565-020-09516-6)

# Overrepresented GO-BP categories

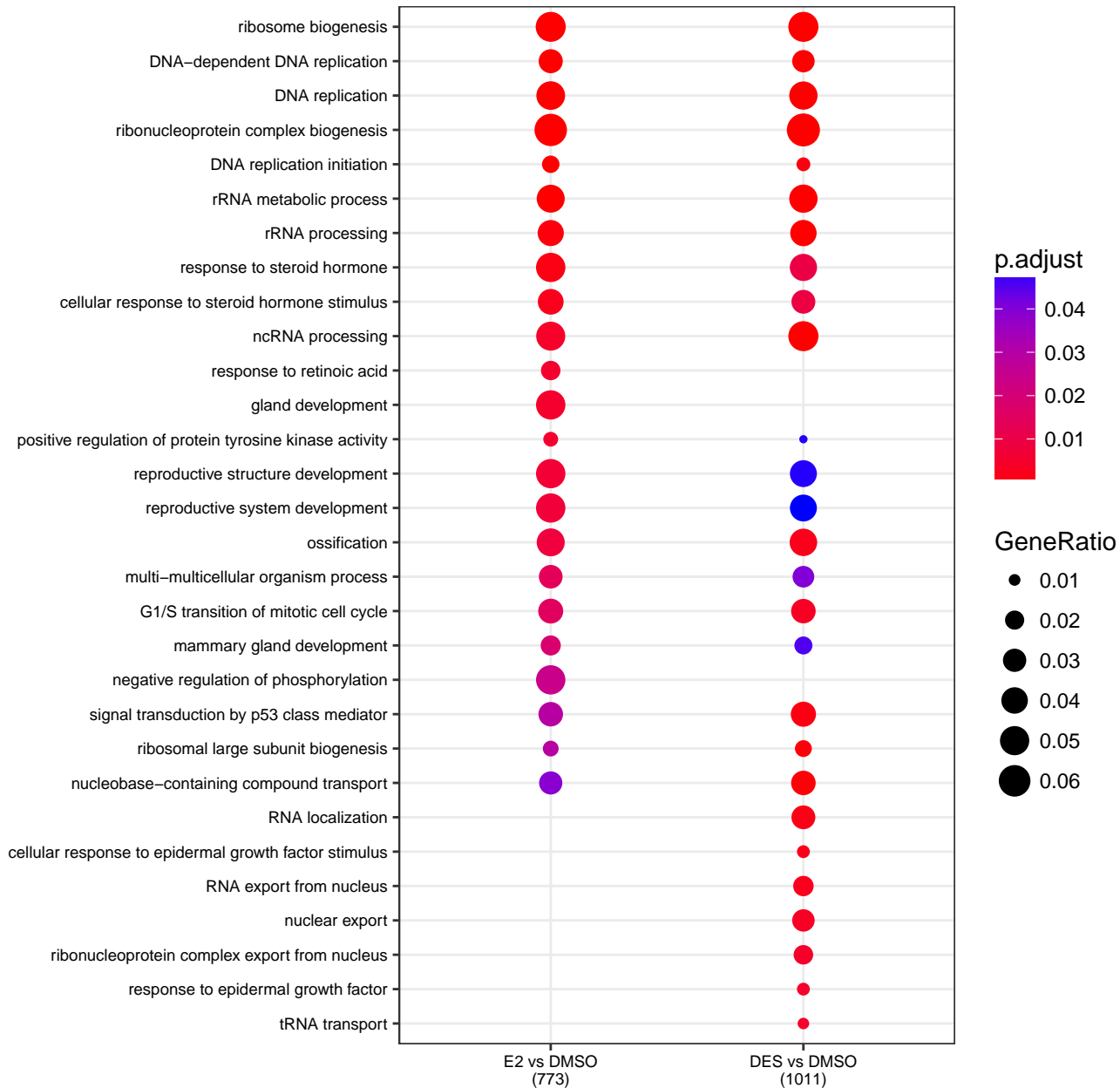

Supplement: Supplementary file 2 — (PDF 7 kb) [file 10565_2020_9516_MOESM2_ESM.pdf]

## Estrogen related receptors pathway

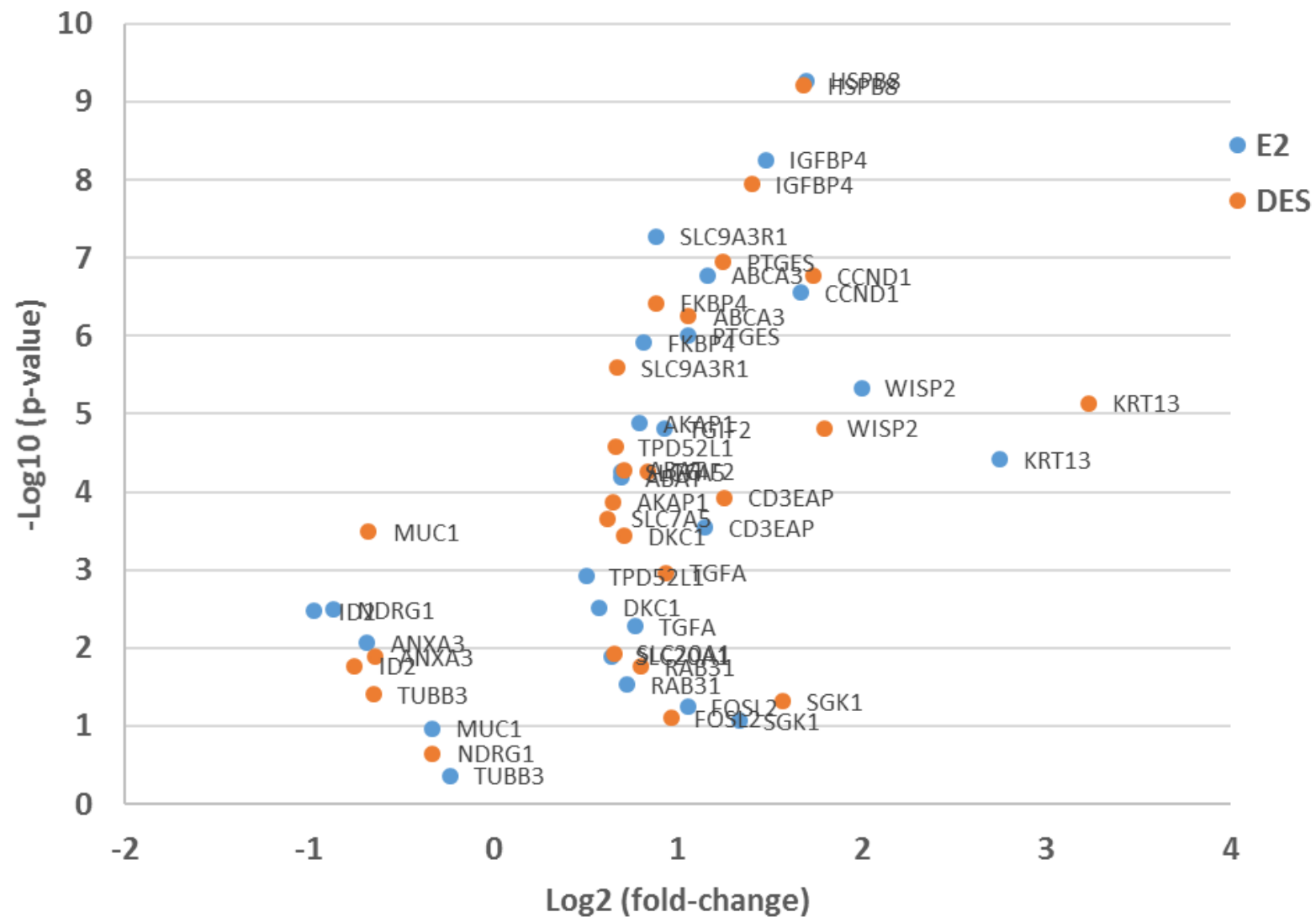

Supplement: Supplementary file 6 — (PDF 42 kb) [file 10565_2020_9516_MOESM6_ESM.pdf]
