# Supplementary material for: Estrogen receptor alpha (ERα)–mediated coregulator binding and gene expression discriminates the toxic ERα agonist diethylstilbestrol (DES) from the endogenous ERα agonist 17β-estradiol (E2)
Source: Cell Biol Toxicol. 2020 Feb 22;36(5):417–35. doi: 10.1007/s10565-020-09516-6 (PMC7505815; doi:10.1007/s10565-020-09516-6)
Supplement: Supplementary file 3 — (PDF 293 kb) [file 10565_2020_9516_MOESM3_ESM.pdf]

Pathway analysis for the genes that expressed by E2 and DES

Pathway name	**set size**	**candidates contained**	**p-value**	**pathway source**
Transcriptional regulation by the AP-2 (TFAP2) family of transcription factors	42	10 (23.8%)	7.58E-07	Reactome
Adipogenesis	131	16 (12.2%)	6.05E-06	Wikipathways
Validated nuclear estrogen receptor alpha network	65	11 (16.9%)	7.67E-06	PID
rRNA processing	67	11 (16.7%)	8.94E-06	Reactome
rRNA modification in the nucleus and cytosol	61	10 (16.7%)	2.29E-05	Reactome
rRNA processing in the nucleus and cytosol	61	10 (16.7%)	2.29E-05	Reactome
Notch-mediated HES/HEY network	51	9 (17.6%)	3.67E-05	PID
HIF-1-alpha transcription factor network	67	10 (14.9%)	6.14E-05	PID
RAF-independent MAPK1/3 activation	23	6 (26.1%)	7.63E-05	Reactome
TGF-beta Signaling Pathway	132	14 (10.6%)	0.000111	Wikipathways
Aryl Hydrocarbon Receptor	46	8 (17.4%)	0.000111	Wikipathways
ID signaling pathway	16	5 (31.2%)	0.000121	Wikipathways
TFAP2 (AP-2) family regulates transcription of growth factors and their receptors	16	5 (31.2%)	0.000121	Reactome
TFAP2 (AP-2) family regulates transcription of other transcription factors	4	3 (75.0%)	0.000138	Reactome
HIF-2-alpha transcription factor network	36	7 (19.4%)	0.000144	PID
Notch Signaling Pathway	61	9 (14.8%)	0.000157	Wikipathways
ID	26	6 (23.1%)	0.00016	NetPath
Ectoderm Differentiation	142	14 (9.9%)	0.000241	Wikipathways
Transcriptional activity of SMAD2-SMAD3-SMAD4 heterotrimer	5	3 (60.0%)	0.000336	Wikipathways
TFAP2 (AP-2) family regulates transcription of cell cycle factors	5	3 (60.0%)	0.000336	Reactome
G1 to S cell cycle control	68	9 (13.2%)	0.000363	Wikipathways
TGF-beta signaling pathway - Homo sapiens (human)	84	10 (11.9%)	0.000416	KEGG
Bladder Cancer	31	6 (19.4%)	0.000446	Wikipathways
IL17 signaling pathway	31	6 (19.4%)	0.000446	Wikipathways
Activation of the TFAP2 (AP-2) family of transcription factors	13	4 (30.8%)	0.00065	Reactome
Signaling by NOTCH1	6	3 (50.0%)	0.000655	Wikipathways

Nuclear Receptors Meta-Pathway	316	22 (7.0%)	0.000767	Wikipathways
Mitotic G1-G1/S phases	92	10 (10.9%)	0.00086	Reactome
TGF-B Signaling in Thyroid Cells for Epithelial-Mesenchymal Transition	14	4 (28.6%)	0.000886	Wikipathways
IL6-mediated signaling events	48	7 (14.6%)	0.000905	PID
tumor suppressor arf inhibits ribosomal biogenesis	24	5 (20.8%)	0.00095	BioCarta
Breast cancer - Homo sapiens (human)	146	13 (8.9%)	0.00105	KEGG
Vitamin D Receptor Pathway	184	15 (8.2%)	0.00105	Wikipathways
TFAP2A acts as a transcriptional repressor during retinoic acid induced cell differentiation	7	3 (42.9%)	0.00112	Reactome
Hepatitis C and Hepatocellular Carcinoma	50	7 (14.0%)	0.00116	Wikipathways
Pancreatic cancer - Homo sapiens (human)	66	8 (12.1%)	0.00138	KEGG
Oncostatin_M	39	6 (15.4%)	0.00158	NetPath
Hippo signaling pathway - Homo sapiens (human)	154	13 (8.5%)	0.00161	KEGG
Spinal Cord Injury	117	11 (9.4%)	0.00162	Wikipathways
Ribosome biogenesis in eukaryotes - Homo sapiens (human)	106	10 (10.0%)	0.00164	KEGG
Neural Crest Differentiation	101	10 (9.9%)	0.00177	Wikipathways
Constitutive Signaling by NOTCH1 HD+PEST Domain Mutants	54	7 (13.0%)	0.00184	Reactome
Signaling by NOTCH1 HD+PEST Domain Mutants in Cancer	54	7 (13.0%)	0.00184	Reactome
Signaling by NOTCH1 in Cancer	54	7 (13.0%)	0.00184	Reactome
Constitutive Signaling by NOTCH1 PEST Domain Mutants	54	7 (13.0%)	0.00184	Reactome
Signaling by NOTCH1 PEST Domain Mutants in Cancer	54	7 (13.0%)	0.00184	Reactome
Bladder cancer - Homo sapiens (human)	41	6 (14.6%)	0.00206	KEGG
Endoderm Differentiation	71	8 (11.3%)	0.00222	Wikipathways
Glucocorticoid Receptor Pathway	71	8 (11.3%)	0.00222	Wikipathways
regulation of map kinase pathways through dual specificity phosphatases	9	3 (33.3%)	0.00256	BioCarta
Interleukin-11 Signaling Pathway	44	6 (13.6%)	0.00298	Wikipathways
Pathways in cancer - Homo sapiens (human)	397	24 (6.0%)	0.00306	KEGG
Validated targets of C-MYC transcriptional repression	75	8 (10.7%)	0.00314	PID
Mammary gland development pathway - Involution (Stage 4 of 4)	10	3 (30.0%)	0.00356	Wikipathways

ATF-2 transcription factor network	61	7 (11.5%)	0.003 72	PID
Aryl Hydrocarbon Receptor Pathway	46	6 (13.0%)	0.003 74	Wikipath ways
Osteoclast differentiation - Homo sapiens (human)	132	11 (8.4%)	0.003 93	KEGG
Regulation of nuclear SMAD2/3 signaling	79	8 (10.3%)	0.004	PID
Integrated Pancreatic Cancer Pathway	170	13 (7.6%)	0.004 05	Wikipath ways
NOTCH1 Intracellular Domain Regulates Transcription	48	6 (12.5%)	0.004 64	Reactome
Interleukin-6 signaling	11	3 (27.3%)	0.004 78	Reactome
VEGFA-VEGFR2 Signaling Pathway	236	16 (6.8%)	0.005	Wikipath ways
HTLV-I infection - Homo sapiens (human)	258	17 (6.6%)	0.005 14	KEGG
Oncostatin M Signaling Pathway	65	7 (10.8%)	0.005 31	Wikipath ways
rac1 cell motility signaling pathway	38	5 (13.9%)	0.006 1	BioCarta
Adenosine P1 receptors	4	2 (50.0%)	0.006 19	Reactome
cyclins and cell cycle regulation	23	4 (17.4%)	0.006 21	BioCarta
Nuclear Receptor transcription pathway	51	6 (11.8%)	0.006 28	Reactome
Cell Cycle	103	9 (8.7%)	0.006 77	Wikipath ways
IL2 signaling events mediated by PI3K	37	5 (13.5%)	0.006 86	PID
Signaling pathways regulating pluripotency of stem cells - Homo sapiens (human)	142	11 (7.7%)	0.007 18	KEGG
Small cell lung cancer - Homo sapiens (human)	86	8 (9.3%)	0.007 23	KEGG
Physiological and Pathological Hypertrophy of the Heart	24	4 (16.7%)	0.007 26	Wikipath ways
mechanism of gene regulation by peroxisome proliferators via ppara	53	6 (11.3%)	0.007 57	BioCarta
Nuclear Receptors	38	5 (13.2%)	0.007 7	Wikipath ways
Cell cycle - Homo sapiens (human)	124	10 (8.1%)	0.007 72	KEGG
Proteoglycans in cancer - Homo sapiens (human)	205	14 (6.8%)	0.007 85	KEGG
Estrogen Receptor Pathway	13	3 (23.1%)	0.007 89	Wikipath ways
melatonin degradation I	13	3 (23.1%)	0.007 89	HumanCy c
NOTCH2 intracellular domain regulates transcription	13	3 (23.1%)	0.007 89	Reactome
G1/S Transition	70	7 (10.0%)	0.007 96	Reactome
IL-7 Signaling Pathway	25	4 (16.0%)	0.008 42	Wikipath ways

Alpha9 beta1 integrin signaling events	25	4 (16.0%)	0.008 42	PID
cell cycle: g1/s check point	25	4 (16.0%)	0.008 42	BioCarta
segmentation clock	25	4 (16.0%)	0.008 42	BioCarta
AP-1 transcription factor network	71	7 (9.9%)	0.008 59	PID
Signaling by NOTCH	108	9 (8.4%)	0.008 63	Reactome
Generic Transcription Pathway	861	41 (4.8%)	0.009 58	Reactome
superpathway of melatonin degradation	14	3 (21.4%)	0.009 8	HumanCy c
Notch signaling pathway	57	6 (10.7%)	0.009 87	PID
Signaling by NOTCH1	74	7 (9.6%)	0.009 95	Reactome
Sulindac Metabolic Pathway	5	2 (40.0%)	0.010 1	Wikipath ways
GRB7 events in ERBB2 signaling	5	2 (40.0%)	0.010 1	Reactome
miR-517 relationship with ARCN1 and USP1	5	2 (40.0%)	0.010 1	Wikipath ways
Transcriptional regulation of pluripotent stem cells	5	2 (40.0%)	0.010 1	Wikipath ways
tRNA modification in the mitochondrion	5	2 (40.0%)	0.010 1	Reactome
DNA Damage Response (only ATM dependent)	110	9 (8.2%)	0.010 3	Wikipath ways
EGF-Ncore	57	6 (10.5%)	0.010 7	Signalink
Interleukin-6 family signaling	27	4 (14.8%)	0.011 1	Reactome
Canonical and Non-canonical Notch signaling	27	4 (14.8%)	0.011 1	Wikipath ways
Signaling by Interleukins	373	21 (5.6%)	0.011 4	Reactome
inactivation of gsk3 by akt causes accumulation of b-catenin in alveolar macrophages	42	5 (11.9%)	0.011 7	BioCarta
IL-2 Signaling Pathway	42	5 (11.9%)	0.011 7	Wikipath ways
Integrated Cancer Pathway	15	3 (20.0%)	0.012	Wikipath ways
ErbB receptor signaling network	15	3 (20.0%)	0.012	PID
Mesodermal Commitment Pathway	153	11 (7.2%)	0.012 2	Wikipath ways
Reelin signaling pathway	28	4 (14.3%)	0.012 6	PID
TGF_beta_Receptor	176	12 (6.9%)	0.012 9	NetPath
IL-6 signaling pathway	43	5 (11.6%)	0.012 9	Wikipath ways
Signaling by PDGF	331	19 (5.8%)	0.012 9	Reactome

Axon guidance - Homo sapiens (human)	177	12 (6.8%)	0.0135	KEGG
Cyclin E associated events during G1/S transition	29	4 (13.8%)	0.0143	Reactome
Amplification and Expansion of Oncogenic Pathways as Metastatic Traits	16	3 (18.8%)	0.0144	Wikipathways
let-7 inhibition of ES cell reprogramming	16	3 (18.8%)	0.0144	Wikipathways
Osteoclast Signaling	16	3 (18.8%)	0.0144	Wikipathways
IL-6 signaling	6	2 (33.3%)	0.0148	INOH
deregulation of cdk5 in alzheimers disease	6	2 (33.3%)	0.0148	BioCarta
E2F transcription factor network	79	7 (8.9%)	0.015	PID
Leptin	62	6 (9.7%)	0.0159	NetPath
p73 transcription factor network	81	7 (8.8%)	0.016	PID
Dopaminergic Neurogenesis	30	4 (13.3%)	0.016	Wikipathways
Interleukin-3, 5 and GM-CSF signaling	226	14 (6.2%)	0.0168	Reactome
Proton Pump Inhibitor Pathway, Pharmacodynamics	46	5 (10.9%)	0.017	PharmGKB
Endochondral Ossification	64	6 (9.5%)	0.0171	Wikipathways
Signaling by EGFR	319	18 (5.7%)	0.0179	Reactome
MAPK1/MAPK3 signaling	206	13 (6.3%)	0.018	Reactome
Wnt Signaling Pathway and Pluripotency	101	8 (7.9%)	0.018	Wikipathways
Presenilin action in Notch and Wnt signaling	47	5 (10.6%)	0.0185	PID
Heart Development	47	5 (10.6%)	0.0185	Wikipathways
Signaling by Leptin	208	13 (6.3%)	0.0194	Reactome
TarBasePathway	18	3 (16.7%)	0.0199	Wikipathways
Monoamine Transport	32	4 (12.5%)	0.02	Wikipathways
wnt signaling pathway	32	4 (12.5%)	0.02	BioCarta
White fat cell differentiation	32	4 (12.5%)	0.02	Wikipathways
miR-148a-miR-31-FIH1-HIF1Î±-Notch signaling in glioblastoma	7	2 (28.6%)	0.0203	Wikipathways
Interleukin receptor SHC signaling	210	13 (6.2%)	0.0208	Reactome
Wnt Signaling Pathway	66	6 (9.1%)	0.021	Wikipathways
Renal cell carcinoma - Homo sapiens (human)	67	6 (9.1%)	0.021	KEGG
NHR	49	5 (10.2%)	0.0218	Signalink
Signaling by NOTCH2	33	4 (12.1%)	0.022	Reactome

			2	
EPHA forward signaling	33	4 (12.1%)	0.0222	PID
EGFR Inhibitor Pathway, Pharmacodynamics	67	6 (9.0%)	0.0225	PharmGKB
Rac1-Pak1-p38-MMP-2 pathway	67	6 (9.0%)	0.0225	Wikipathways
VEGFR2 mediated cell proliferation	213	13 (6.1%)	0.023	Reactome
Phase 4 - resting membrane potential	19	3 (15.8%)	0.0231	Reactome
VEGFA-VEGFR2 Pathway	282	16 (5.7%)	0.0235	Reactome
EPHA-mediated growth cone collapse	34	4 (11.8%)	0.0245	Reactome
Resolution of D-loop Structures through Holliday Junction Intermediates	35	4 (11.8%)	0.0245	Reactome
Downstream signal transduction	307	17 (5.6%)	0.0246	Reactome
TP53 Regulates Transcription of Cell Cycle Genes	51	5 (9.8%)	0.0255	Reactome
Interleukin-2 signaling	217	13 (6.0%)	0.0263	Reactome
Synthesis of epoxy (EET) and dihydroxyeicosatrienoic acids (DHET)	8	2 (25.0%)	0.0265	Reactome
Interleukin-17 signaling	8	2 (25.0%)	0.0265	Reactome
IL-6-type cytokine receptor ligand interactions	20	3 (15.0%)	0.0265	Reactome
MAPK family signaling cascades	240	14 (5.9%)	0.0266	Reactome
Androgen receptor signaling pathway	89	7 (7.9%)	0.027	Wikipathways
RET signaling	219	13 (6.0%)	0.0281	Reactome
Signaling by VEGF	290	16 (5.5%)	0.0295	Reactome
Resolution of D-Loop Structures	37	4 (11.1%)	0.0296	Reactome
downregulated of mta-3 in er-negative breast tumors	21	3 (14.3%)	0.0302	BioCarta
Methionine De Novo and Salvage Pathway	21	3 (14.3%)	0.0302	Wikipathways
Cytokine Signaling in Immune system	487	24 (4.9%)	0.0309	Reactome
RAF/MAP kinase cascade	200	12 (6.0%)	0.0316	Reactome
SHC1 events in EGFR signaling	200	12 (6.0%)	0.0316	Reactome
SOS-mediated signalling	200	12 (6.0%)	0.0316	Reactome
GRB2 events in EGFR signaling	200	12 (6.0%)	0.0316	Reactome
IL2-mediated signaling events	54	5 (9.3%)	0.0317	PID
Circadian Clock	38	4 (10.8%)	0.032	Reactome

			3	
Activation of ATR in response to replication stress	37	4 (10.8%)	0.032 3	Reactome
Chronic myeloid leukemia - Homo sapiens (human)	73	6 (8.2%)	0.032 6	KEGG
Adrenoceptors	9	2 (22.2%)	0.033 3	Reactome
Synthesis of (16-20)-hydroxyeicosatetraenoic acids (HETE)	9	2 (22.2%)	0.033 3	Reactome
MAPK1 (ERK2) activation	9	2 (22.2%)	0.033 3	Reactome
Metabolism of ingested SeMet, Sec, MeSec into H2Se	9	2 (22.2%)	0.033 3	Reactome
TGF-beta Receptor Signaling	55	5 (9.1%)	0.034	Wikipath ways
ErbB Signaling Pathway	55	5 (9.1%)	0.034	Wikipath ways
Signalling to p38 via RIT and RIN	204	12 (5.9%)	0.036	Reactome
ARMS-mediated activation	204	12 (5.9%)	0.036	Reactome
Viral carcinogenesis - Homo sapiens (human)	203	12 (5.9%)	0.036	KEGG
Regulation of lipolysis in adipocytes - Homo sapiens (human)	56	5 (8.9%)	0.036 4	KEGG
Non-small cell lung cancer - Homo sapiens (human)	56	5 (8.9%)	0.036 4	KEGG
Sudden Infant Death Syndrome (SIDS) Susceptibility Pathways	159	10 (6.3%)	0.037 1	Wikipath ways
Frs2-mediated activation	205	12 (5.9%)	0.037 2	Reactome
Cyclin D associated events in G1	39	4 (10.3%)	0.038 3	Reactome
G1 Phase	39	4 (10.3%)	0.038 3	Reactome
Signaling events regulated by Ret tyrosine kinase	39	4 (10.3%)	0.038 3	PID
O-glycosylation of TSR domain-containing proteins	39	4 (10.3%)	0.038 3	Reactome
IL11	23	3 (13.0%)	0.038 4	NetPath
Sympathetic Nerve Pathway (Neuroeffector Junction)	23	3 (13.0%)	0.038 4	PharmGK B
ctcf: first multivalent nuclear factor	23	3 (13.0%)	0.038 4	BioCarta
Prolactin Signaling Pathway	76	6 (7.9%)	0.038 6	Wikipath ways
Acute myeloid leukemia - Homo sapiens (human)	57	5 (8.8%)	0.038 8	KEGG
Prolonged ERK activation events	207	12 (5.8%)	0.039 6	Reactome
NCAM signaling for neurite out-growth	231	13 (5.7%)	0.040 7	Reactome
Organic cation transport	11	2 (20.0%)	0.040 7	Reactome
MAPK3 (ERK1) activation	10	2 (20.0%)	0.040 7	Reactome

LIF signaling	10	2 (20.0%)	0.0407	INOH
methionine salvage cycle III	10	2 (20.0%)	0.0407	HumanCyc
overview of telomerase protein component gene htert transcriptional regulation	10	2 (20.0%)	0.0407	BioCarta
Signaling by FGFR3 fusions in cancer	10	2 (20.0%)	0.0407	Reactome
EV release from cardiac cells and their functional effects	10	2 (20.0%)	0.0407	Wikipathways
RORA activates gene expression	10	2 (20.0%)	0.0407	Reactome
Negative regulation of activity of TFAP2 (AP-2) family transcription factors	10	2 (20.0%)	0.0407	Reactome
IL6	77	6 (7.8%)	0.0408	NetPath
Negative regulation of MAPK pathway	40	4 (10.0%)	0.0415	Reactome
IL-5 Signaling Pathway	40	4 (10.0%)	0.0415	Wikipathways
tRNA modification in the nucleus and cytosol	40	4 (10.0%)	0.0415	Reactome
Signalling to RAS	209	12 (5.8%)	0.0421	Reactome
Glycosaminoglycan biosynthesis - heparan sulfate / heparin - Homo sapiens (human)	24	3 (12.5%)	0.0428	KEGG
Kit receptor signaling pathway	59	5 (8.5%)	0.044	Wikipathways
SHP2 signaling	59	5 (8.5%)	0.044	PID
Preimplantation Embryo	59	5 (8.5%)	0.044	Wikipathways
G2/M Checkpoints	121	8 (6.7%)	0.0442	Reactome
Cell Differentiation - meta	60	5 (8.3%)	0.0468	Wikipathways
Rap1 signaling pathway - Homo sapiens (human)	212	12 (5.7%)	0.0475	KEGG
Nucleotide GPCRs	11	2 (18.2%)	0.0487	Wikipathways
Signaling mediated by p38-gamma and p38-delta	12	2 (18.2%)	0.0487	PID
il22 soluble receptor signaling pathway	11	2 (18.2%)	0.0487	BioCarta
cxcr4 signaling pathway	11	2 (18.2%)	0.0487	BioCarta
Signaling by FGFR4 in disease	11	2 (18.2%)	0.0487	Reactome
cardiac protection against ros	12	2 (18.2%)	0.0487	BioCarta
POU5F1 (OCT4), SOX2, NANOG repress genes related to differentiation	11	2 (18.2%)	0.0487	Reactome
Axon guidance	487	23 (4.8%)	0.0491	Reactome
Developmental Biology	748	33 (4.4%)	0.0494	Reactome

Coregulation of Androgen receptor activity	61	5 (8.2%)	0.049 7	PID
Notch	62	5 (8.2%)	0.049 7	NetPath
