# Supplementary material for: Estrogen receptor alpha (ERα)–mediated coregulator binding and gene expression discriminates the toxic ERα agonist diethylstilbestrol (DES) from the endogenous ERα agonist 17β-estradiol (E2)
Source: Cell Biol Toxicol. 2020 Feb 22;36(5):417–35. doi: 10.1007/s10565-020-09516-6 (PMC7505815; doi:10.1007/s10565-020-09516-6)
Supplement: Supplementary file 4 — (PDF 247 kb) [file 10565_2020_9516_MOESM4_ESM.pdf]

Pathway analyses of the genes that expressed by DES only

Pathway name	set size	candidates contained	p-value	pathway source
Electron Transport Chain	103	12 (11.7%)	1.43E-05	Wikipathways
Oxidative phosphorylation - Homo sapiens (human)	133	13 (9.8%)	4.23E-05	KEGG
Parkinson,s disease - Homo sapiens (human)	142	13 (9.2%)	8.36E-05	KEGG
Transmembrane transport of small molecules	628	32 (5.1%)	0.0002	Reactome
Respiratory electron transport, ATP synthesis by chemiosmotic coupling, and heat production by uncoupling proteins.	126	11 (8.8%)	0.000411	Reactome
The citric acid (TCA) cycle and respiratory electron transport	171	13 (7.6%)	0.000498	Reactome
Oxidative phosphorylation	61	7 (11.5%)	0.000985	Wikipathways
Respiratory electron transport	103	9 (8.8%)	0.00134	Reactome
Exercise-induced Circadian Regulation	48	6 (12.5%)	0.00144	Wikipathways
Stimuli-sensing channels	104	9 (8.7%)	0.00153	Reactome
Multifunctional anion exchangers	10	3 (30.0%)	0.00182	Reactome
Mineral absorption - Homo sapiens (human)	52	6 (11.5%)	0.00219	KEGG
Allograft rejection - Homo sapiens (human)	38	5 (13.5%)	0.00254	KEGG
Graft-versus-host disease - Homo sapiens (human)	41	5 (12.2%)	0.00401	KEGG
Endosomal/Vacuolar pathway	13	3 (23.1%)	0.0041	Reactome
Glycerolipid metabolism - Homo sapiens (human)	59	6 (10.2%)	0.00417	KEGG
Neural Crest Differentiation	101	8 (7.9%)	0.00476	Wikipathways
Type I diabetes mellitus - Homo sapiens (human)	43	5 (11.6%)	0.00493	KEGG
GPR40 PATHWAY	14	3 (21.4%)	0.00512	Wikipathways
Insulin secretion - Homo sapiens (human)	85	7 (8.2%)	0.00657	KEGG
Transport of inorganic cations/anions and amino acids/oligopeptides	107	8 (7.5%)	0.00673	Reactome
Alcoholism - Homo sapiens (human)	180	11 (6.1%)	0.00743	KEGG
Ion channel transport	209	12 (5.7%)	0.00856	Reactome
mechanism of acetaminophen activity and toxicity	6	2 (33.3%)	0.00943	BioCarta
Autoimmune thyroid disease - Homo sapiens (human)	53	5 (9.6%)	0.011	KEGG
HDACs deacetylate histones	94	7 (7.4%)	0.0112	Reactome
Formation of the beta-catenin:TCF transactivating complex	95	7 (7.4%)	0.0112	Reactome
NrCAM interactions	7	2 (28.6%)	0.013	Reactome
Proton-coupled monocarboxylate transport	7	2 (28.6%)	0.013	Reactome
Binding of TCF/LEF:CTNNB1 to target gene promoters	7	2 (28.6%)	0.013	Reactome
Alzheimer,s disease - Homo sapiens (human)	171	10 (5.8%)	0.0139	KEGG
Glutathione metabolism	20	3 (15.0%)	0.0143	Wikipathways
Wnt-beta-catenin Signaling Pathway in Leukemia	20	3 (15.0%)	0.0143	Wikipathways
RNA degradation - Homo sapiens (human)	77	6 (7.8%)	0.0149	KEGG

ERCC6 (CSB) and EHMT2 (G9a) positively regulate rRNA expression	79	6 (7.7%)	0.0158	Reactome
Complex I biogenesis	57	5 (8.8%)	0.016	Reactome
CDC6 association with the ORC:origin complex	8	2 (25.0%)	0.017	Reactome
Recognition and association of DNA glycosylase with site containing an affected pyrimidine	8	2 (25.0%)	0.017	Reactome
Cleavage of the damaged pyrimidine	8	2 (25.0%)	0.017	Reactome
Depyrimidination	8	2 (25.0%)	0.017	Reactome
Histidine catabolism	8	2 (25.0%)	0.017	Reactome
Allograft Rejection	80	6 (7.5%)	0.0177	Wikipathways
Phagosome - Homo sapiens (human)	154	9 (5.9%)	0.0185	KEGG
Homologous recombination - Homo sapiens (human)	41	4 (9.8%)	0.0213	KEGG
sphingosine and sphingosine-1-phosphate metabolism	9	2 (22.2%)	0.0215	HumanCyc
Antigen Presentation: Folding, assembly and peptide loading of class I MHC	24	3 (12.5%)	0.0235	Reactome
Histidine metabolism - Homo sapiens (human)	24	3 (12.5%)	0.0235	KEGG
Meiotic recombination	65	5 (7.8%)	0.0252	Reactome
TRP channels	25	3 (12.0%)	0.0262	Reactome
Glycogen synthesis	10	2 (20.0%)	0.0264	Reactome
Base-Excision Repair, AP Site Formation	10	2 (20.0%)	0.0264	Reactome
RNA Polymerase I Promoter Opening	66	5 (7.7%)	0.0267	Reactome
Tacrolimus/Cyclosporine Pathway, Pharmacodynamics	44	4 (9.1%)	0.0269	PharmGKB
Integrins in angiogenesis	66	5 (7.6%)	0.0283	PID
DNA methylation	68	5 (7.5%)	0.03	Reactome
Signaling by Rho GTPases	434	18 (4.2%)	0.0315	Reactome
Gamma-glutamyl-transpeptidase deficiency	11	2 (18.2%)	0.0317	SMPDB
5-oxoprolinase deficiency	11	2 (18.2%)	0.0317	SMPDB
Gamma-Glutamyltransferase Deficiency	11	2 (18.2%)	0.0317	SMPDB
Glutathione Metabolism	11	2 (18.2%)	0.0317	SMPDB
Glutathione Synthetase Deficiency	11	2 (18.2%)	0.0317	SMPDB
5-Oxoprolinuria	11	2 (18.2%)	0.0317	SMPDB
Spinal Cord Injury	117	7 (6.0%)	0.0329	Wikipathways
Activated PKN1 stimulates transcription of AR (androgen receptor) regulated genes KLK2 and KLK3	70	5 (7.2%)	0.0335	Reactome
Synthesis of IP3 and IP4 in the cytosol	28	3 (10.7%)	0.0353	Reactome
Resolution of D-loop Structures through Synthesis-Dependent Strand Annealing (SDSA)	28	3 (10.7%)	0.0353	Reactome
SLC-mediated transmembrane transport	286	13 (4.6%)	0.0356	Reactome
il-2 receptor beta chain in t cell activation	48	4 (8.3%)	0.0356	BioCarta
Longevity regulating pathway - Homo sapiens (human)	94	6 (6.4%)	0.0357	KEGG
Bile secretion - Homo sapiens (human)	71	5 (7.0%)	0.0372	KEGG
SIRT1 negatively regulates rRNA Expression	72	5 (7.0%)	0.0372	Reactome
Glycerophospholipid metabolism - Homo sapiens (human)	95	6 (6.3%)	0.0373	KEGG
antigen processing and presentation	12	2 (16.7%)	0.0374	BioCarta
Generic Transcription Pathway	861	31 (3.6%)	0.038	Reactome

Cocaine addiction - Homo sapiens (human)	49	4 (8.2%)	0.038	KEGG
Antigen processing-Cross presentation	49	4 (8.2%)	0.038	Reactome
Dectin-2 family	29	3 (10.3%)	0.0386	Reactome
triacylglycerol biosynthesis	29	3 (10.3%)	0.0386	HumanCyc
Axon guidance - Homo sapiens (human)	177	9 (5.1%)	0.0405	KEGG
TP53 Regulates Transcription of Cell Cycle Genes	13	2 (15.4%)	0.0435	Wikipathways
Familial lipoprotein lipase deficiency	13	2 (15.4%)	0.0435	SMPDB
Glycerolipid Metabolism	13	2 (15.4%)	0.0435	SMPDB
Glycerol Kinase Deficiency	13	2 (15.4%)	0.0435	SMPDB
D-glyceric acidura	13	2 (15.4%)	0.0435	SMPDB
multiple antiapoptotic pathways from igf-1r signaling lead to bad phosphorylation	13	2 (15.4%)	0.0435	BioCarta
Methotrexate Pathway, Pharmacokinetics	13	2 (15.4%)	0.0435	PharmGKB
Transcriptional misregulation in cancer - Homo sapiens (human)	180	9 (5.0%)	0.0443	KEGG
Fatty acid, triacylglycerol, and ketone body metabolism	153	8 (5.3%)	0.045	Reactome
role of mef2d in t-cell apoptosis	31	3 (9.7%)	0.0458	BioCarta
Potassium Channels	100	6 (6.0%)	0.046	Reactome
PRC2 methylates histones and DNA	77	5 (6.6%)	0.0476	Reactome
Condensation of Prophase Chromosomes	77	5 (6.6%)	0.0476	Reactome
CD4 T cell receptor signaling-JNK cascade	53	4 (7.5%)	0.0485	INOH
Oxidative Stress Induced Senescence	129	7 (5.5%)	0.0495	Reactome
Antigen processing and presentation - Homo sapiens (human)	77	5 (6.5%)	0.0499	KEGG
Irinotecan Pathway	14	2 (14.3%)	0.0499	Wikipathways
CDT1 association with the CDC6:ORC:origin complex	14	2 (14.3%)	0.0499	Reactome
Repression of WNT target genes	14	2 (14.3%)	0.0499	Reactome
