# Supplementary material for: Estrogen receptor alpha (ERα)–mediated coregulator binding and gene expression discriminates the toxic ERα agonist diethylstilbestrol (DES) from the endogenous ERα agonist 17β-estradiol (E2)
Source: Cell Biol Toxicol. 2020 Feb 22;36(5):417–35. doi: 10.1007/s10565-020-09516-6 (PMC7505815; doi:10.1007/s10565-020-09516-6)
Supplement: Supplementary file 5 — (PDF 226 kb) [file 10565_2020_9516_MOESM5_ESM.pdf]

Pathway analyses of the genes that expressed by E2 only

Pathway name	Set size	Candidate conttained	p-value	Pathway source
HDMs demethylate histones	52	5 (9.6%)	0.00176	Reactome
BMP Signalling Pathway	19	3 (15.8%)	0.00373	HumanCyc
Lipid storage and perilipins in skeletal muscle	6	2 (33.3%)	0.00405	Wikipathways
Hormone-sensitive lipase (HSL)-mediated triacylglycerol hydrolysis	20	3 (15.0%)	0.00434	Reactome
Signaling by BMP	22	3 (13.6%)	0.00572	Reactome
Collagen biosynthesis and modifying enzymes	70	5 (7.1%)	0.00643	Reactome
Glycosphingolipid metabolism	46	4 (8.7%)	0.00733	Reactome
Vitamin C (ascorbate) metabolism	8	2 (25.0%)	0.00739	Reactome
Collagen chain trimerization	47	4 (8.5%)	0.00791	Reactome
Synaptic Vesicle Pathway	51	4 (7.8%)	0.0105	Wikipathways
Resolution of D-loop Structures through Synthesis-Dependent Strand Annealing (SDSA)	28	3 (10.7%)	0.0113	Reactome
Interaction between L1 and Ankyrins	30	3 (10.0%)	0.0137	Reactome
Ligand-independent caspase activation via DCC	11	2 (18.2%)	0.014	Reactome
BMP2 signaling TGF-beta MV	56	4 (7.1%)	0.0145	INOH
Ovarian Infertility Genes	31	3 (9.7%)	0.0149	Wikipathways
Mucin type O-glycan biosynthesis - Homo sapiens (human)	31	3 (9.7%)	0.0149	KEGG
Viral myocarditis - Homo sapiens (human)	59	4 (6.9%)	0.0163	KEGG
P2Y receptors	12	2 (16.7%)	0.0167	Reactome
Bone Morphogenic Protein (BMP) Signalling and Regulation	12	2 (16.7%)	0.0167	Wikipathways
Protein digestion and absorption - Homo sapiens (human)	90	5 (5.6%)	0.0179	KEGG
BMP signaling Dro	34	3 (8.8%)	0.0192	INOH
Resolution of D-loop Structures through Holliday Junction Intermediates	35	3 (8.8%)	0.0192	Reactome
Depolarization of the Presynaptic Terminal Triggers the Opening of Calcium Channels	13	2 (15.4%)	0.0195	Reactome
The activation of arylsulfatases	13	2 (15.4%)	0.0195	Reactome
Collagen formation	94	5 (5.3%)	0.0211	Reactome
Synaptic vesicle cycle - Homo sapiens (human)	63	4 (6.3%)	0.0215	KEGG
Resolution of D-Loop Structures	37	3 (8.3%)	0.0223	Reactome
IL5-mediated signaling events	14	2 (14.3%)	0.0225	PID
TP53 regulates transcription of several additional cell death genes whose specific roles in p53-dependent apoptosis remain uncertain	14	2 (14.3%)	0.0225	Reactome
triacylglycerol degradation	14	2 (14.3%)	0.0225	HumanCyc
Synthesis of PE	14	2 (14.3%)	0.0225	Reactome

Adipogenesis	131	6 (4.6%)	0.0233	Wikipathways
HIF-1-alpha transcription factor network	67	4 (6.0%)	0.0262	PID
Metabolism of nucleotides	100	5 (5.0%)	0.0268	Reactome
Nucleotide-like (purinergic) receptors	16	2 (12.5%)	0.029	Reactome
Deregulation of Rab and Rab Effector Genes in Bladder Cancer	16	2 (12.5%)	0.029	Wikipathways
BMP receptor signaling	41	3 (7.3%)	0.0314	PID
Bile Acid Biosynthesis	17	2 (11.8%)	0.0325	SMPDB
27-Hydroxylase Deficiency	17	2 (11.8%)	0.0325	SMPDB
Congenital Bile Acid Synthesis Defect Type II	17	2 (11.8%)	0.0325	SMPDB
Cerebrotendinous Xanthomatosis (CTX)	17	2 (11.8%)	0.0325	SMPDB
Zellweger Syndrome	17	2 (11.8%)	0.0325	SMPDB
Familial Hypercholanemia (FHCA)	17	2 (11.8%)	0.0325	SMPDB
Congenital Bile Acid Synthesis Defect Type III	17	2 (11.8%)	0.0325	SMPDB
Primary bile acid biosynthesis - Homo sapiens (human)	17	2 (11.8%)	0.0325	KEGG
TGF-Core	42	3 (7.1%)	0.0334	Signalink
Protein-protein interactions at synapses	73	4 (5.5%)	0.0345	Reactome
Transmembrane transport of small molecules	628	17 (2.7%)	0.0354	Reactome
Homologous DNA Pairing and Strand Exchange	43	3 (7.0%)	0.0354	Reactome
JAK-STAT	43	3 (7.0%)	0.0354	Wikipathways
Estrogen metabolism	18	2 (11.1%)	0.0361	Wikipathways
ABC transporters in lipid homeostasis	18	2 (11.1%)	0.0361	Reactome
Other glycan degradation - Homo sapiens (human)	18	2 (11.1%)	0.0361	KEGG
Lipid digestion, mobilization, and transport	110	5 (4.5%)	0.0381	Reactome
PRC2 methylates histones and DNA	77	4 (5.3%)	0.0391	Reactome
Glucose transport	45	3 (6.7%)	0.0398	Reactome
Growth hormone receptor signaling	19	2 (10.5%)	0.04	Reactome
HH-Core	19	2 (10.5%)	0.04	Signalink
Calcium Regulation in the Cardiac Cell	149	6 (4.0%)	0.0402	Wikipathways
RNA degradation - Homo sapiens (human)	77	4 (5.2%)	0.0408	KEGG
Globo Sphingolipid Metabolism	20	2 (10.0%)	0.0439	Wikipathways
sprouty regulation of tyrosine kinase signals	20	2 (10.0%)	0.0439	BioCarta
Hippo signaling pathway - Homo sapiens (human)	154	6 (3.9%)	0.0447	KEGG
Allograft Rejection	80	4 (5.0%)	0.0458	Wikipathways
Asymmetric localization of PCP proteins	21	2 (9.5%)	0.048	Reactome
Toxoplasmosis - Homo sapiens (human)	118	5 (4.2%)	0.0491	KEGG
