# Supplementary material for: Estrogen receptor alpha (ERα)–mediated coregulator binding and gene expression discriminates the toxic ERα agonist diethylstilbestrol (DES) from the endogenous ERα agonist 17β-estradiol (E2)
Source: Cell Biol Toxicol. 2020 Feb 22;36(5):417–35. doi: 10.1007/s10565-020-09516-6 (PMC7505815; doi:10.1007/s10565-020-09516-6)
Supplement: Supplementary file 7 — (PDF 275 kb) [file 10565_2020_9516_MOESM7_ESM.pdf]

The genes that are validated in MCF-7 cells

Histone modification- related genes

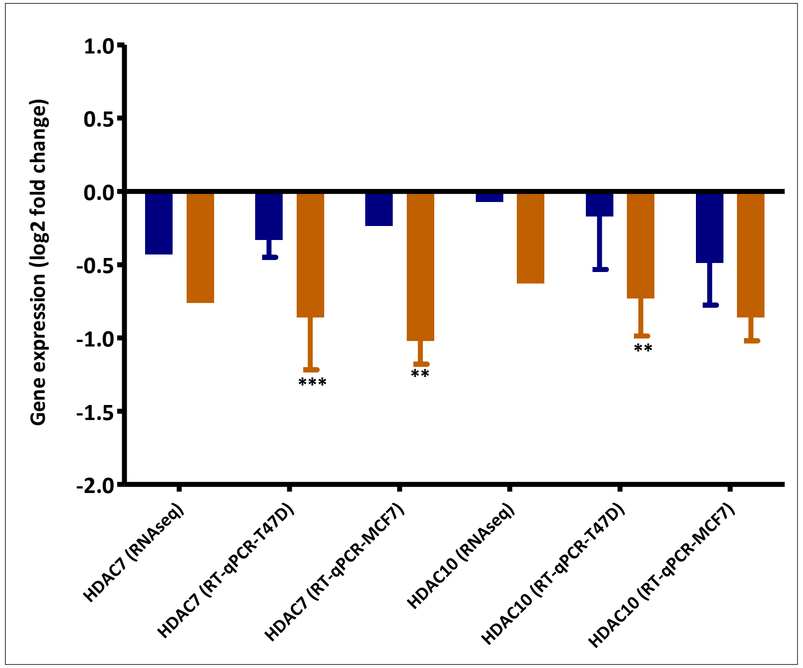

RNA-seq and RT-qPCR characterization of the effects of DES (orange) and E2 (blue) on gene expression associated with RAR pathways. For RT-qPCR results are expressed as log2 fold changes in relation to the solvent control, bars represent average ± SEM from at least three independent experiments. For statistical analysis of the RT-qPCR data multiple paired t-tests were performed and differences were considered significant if p-value <0.05

RAR-related genes

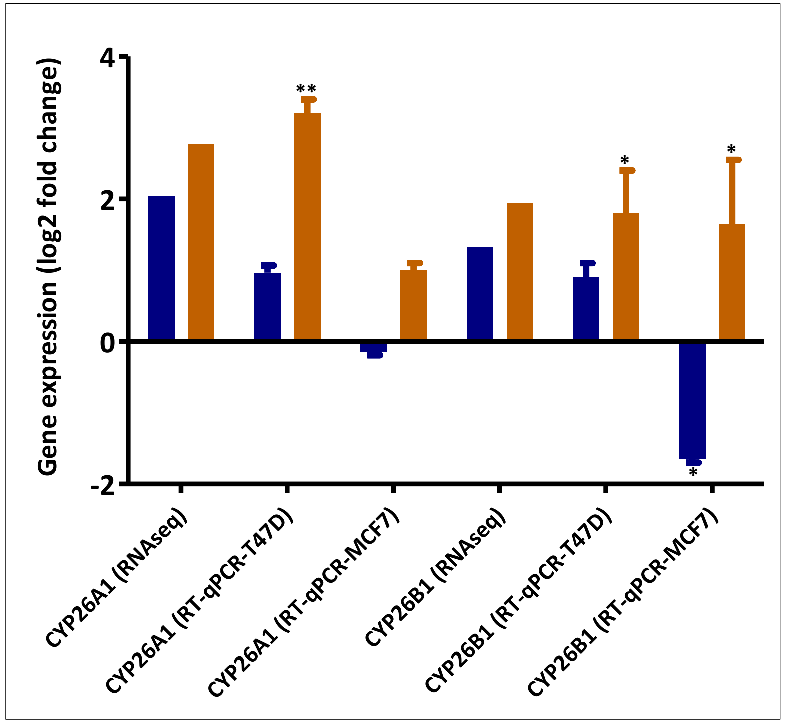

RNA-seq and RT-qPCR characterization of the effects of DES (orange) and E2 (blue) on gene expression associated with RAR pathways. For RT-qPCR results are expressed as log2 fold changes in relation to the solvent control, bars represent average ± SEM from at least three independent experiments. For statistical analysis of the RT-qPCR data multiple paired t-tests were performed and differences were considered significant if p-value <0.05

ER-related genes

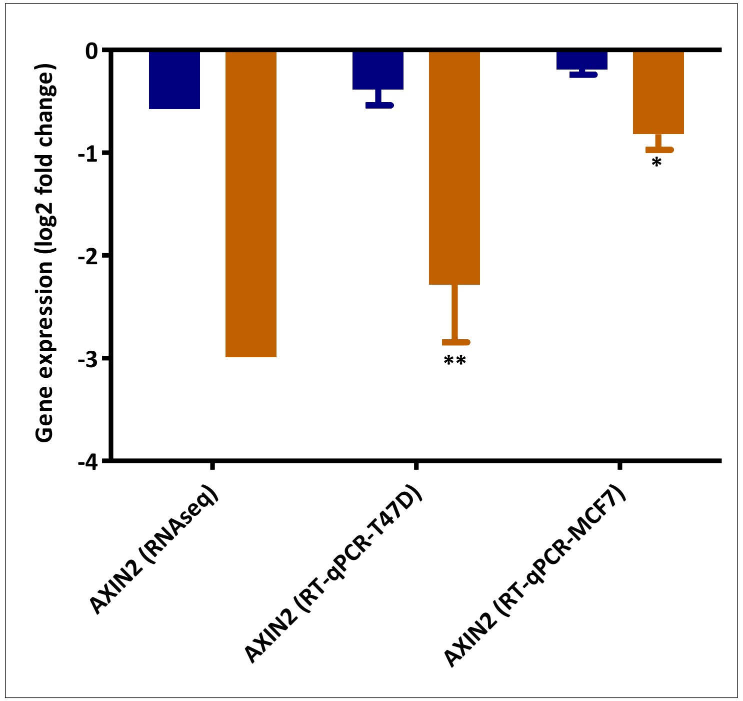

RNA-seq and RT-qPCR characterization of the effects of DES (orange) and E2 (blue) on gene expression associated with RAR pathways. For RT-qPCR results are expressed as log2 fold changes in relation to the solvent control, bars represent average ± SEM from at least three independent experiments. For statistical analysis of the RT-qPCR data multiple paired t-tests were performed and differences were considered significant if p-value <0.05
